# Microcantilever: Dynamical Response for Mass Sensing and Fluid Characterization

**DOI:** 10.3390/s21010115

**Published:** 2020-12-27

**Authors:** João Mouro, Rui Pinto, Paolo Paoletti, Bruno Tiribilli

**Affiliations:** 1 Institute for Complex Systems (ISC-CNR), National Research Council, Via Madonna del Piano 10, 50019 Florence, Italy; bruno.tiribilli@isc.cnr.it; 2 INESC—Microsystems e Nanotecnologies, Rua Alves Redol, 9, 1000-029 Lisbon, Portugal; ruimrpinto@yahoo.com; 3 School of Engineering, University of Liverpool, Liverpool L69 3GH, UK; P.Paoletti@liverpool.ac.uk

**Keywords:** microcantilever, mass sensing, rheology sensing, noise, non-Newtonian viscoelastic fluids

## Abstract

A microcantilever is a suspended micro-scale beam structure supported at one end which can bend and/or vibrate when subjected to a load. Microcantilevers are one of the most fundamental miniaturized devices used in microelectromechanical systems and are ubiquitous in sensing, imaging, time reference, and biological/biomedical applications. They are typically built using micro and nanofabrication techniques derived from the microelectronics industry and can involve microelectronics-related materials, polymeric materials, and biological materials. This work presents a comprehensive review of the rich dynamical response of a microcantilever and how it has been used for measuring the mass and rheological properties of Newtonian/non-Newtonian fluids in real time, in ever-decreasing space and time scales, and with unprecedented resolution.

## 1. Introduction

Fluids play a key role in many sensing applications based on microelectromechanical systems (MEMS), being either the substance to be tested (when measuring rheological properties) or the support environment used to keep the substance of interest in its native or physiological state (when detecting proteins, DNA, or other analytes in a solution). The understanding of the interaction of the sensor with the surrounding medium is a key topic in the process of measuring the mass of analytes with extremely high—potentially single-molecule—accuracy, and when using MEMS sensors to study the rheology of simple and complex fluids. Such wide sensing applications span the fields of the food and process industry, environmental monitoring, healthcare, microfluidics, and others. Several of these problems still do not have an adequate solution, but huge progress has been made in the last two decades by exploiting microcantilevers, miniaturized beams supported at one end. Microcantilevers are a traditional but crucial MEMS design used for sensing, imaging, and time-keeping applications.

This paper presents a thorough review of how the complex dynamical response of the microcantilever excited by a periodic force and interacting with the surrounding environment can be used for mass and rheological sensing. Some examples of the latest and more significant results are provided, and the physical principles behind the applications are discussed. The work is organized as follows. In Section 2, the mechanics and dynamical response of the microcantilever are presented. The goal is to set the theoretical framework, consisting of some classical models, to determine the resonance frequencies and quality factors of each resonant mode in different media, which will be used throughout the rest of the work. Section 3 contains a discussion of the mechanisms used to excite the dynamical response of the probe, including open and closed-loop schemes typically found in sensing applications. Particular focus is dedicated to feedback loops, which have shown significant promise and exciting new results in recent years, by providing relevant examples that are currently being developed and studied to improve the performance of microcantilever-based sensors. A thorough discussion of noise measurements and mechanisms follows. This aspect is often overlooked in the literature, but it is a fundamental feature to consider when designing a sensor. Section 4 is dedicated to discussing the principle of mass sensing, sensitivities, and limits of detection and shows some examples of recent major achievements in this area. Finally, in Section 5 it is shown how the microcantilever can be used as a rheological sensor to measure the properties of Newtonian and non-Newtonian fluids in real time. Section 6 summarizes current open challenges and presents an outlook on future opportunities for microcantilever-based rheology and mass sensors with the aim of stimulating further research in this field.

## 2. Cantilever Mechanics and Dynamical Response

### 2.1. Euler–Bernoulli Beam

**Figure 1:**
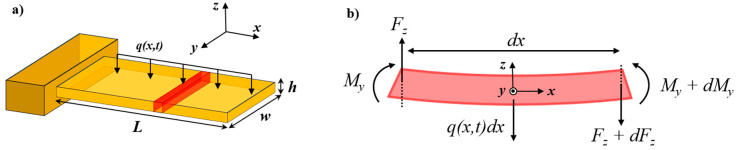
(**a**) Schematic of a cantilever beam of length *L*, width *w*, and thickness *h*. The cantilever is subjected to a distributed time-changing load per unit length, *q*(*x*, *t*); (**b**) longitudinal cross section of an infinitesimal element *dx* of the same cantilever (red part highlighted in (**a**)), where the shear forces and bending moments act.

The flexural vibration of a thin uniform beam can be described by the well-known Euler–Bernoulli beam theory. This model is based on four main assumptions: (i) cantilever aspect ratios L/w and L/h are large (L≫w,h), (ii) deflections are small when compared to the beam dimensions, (iii) the cantilever material is linear elastic and homogeneous, and (iv) no dissipation occurs during deformation. The schematic of such a cantilever subjected to an external force per unit length q(x,t) is shown in Figure 1a. The external load is responsible for the existence of shear forces $F_{z}$ and bending moments $M_{y}$ that act on each element of the beam of infinitesimal length *dx*, as shown in Figure 1b.

Balancing the forces (*z*-direction) and the bending moments (*y*-direction) acting on each infinitesimal element of the device and neglecting higher order terms leads to the following equations (see Figure 1b) [1,2]:

(1) $$\{\begin{array}{c} F_{z}+dF_{z}-F_{z}+q\left(x,t\right)dx=\rho Adx\frac{\partial^{2}W\left(x,t\right)}{\partial t^{2}}\\ M_{y}+dM_{y}-M_{y}-F_{z}dx+q\left(x,t\right)dx\frac{dx}{2}=0 \end{array}\overset{}{\Rightarrow }\{\begin{array}{c} \frac{dF_{z}}{dx}+q\left(x,t\right)=\rho A\frac{\partial^{2}W\left(x,t\right)}{\partial t^{2}}\\ \frac{dM_{y}}{dx}=F_{z} \end{array},$$

where W(x,t) is the time-varying deflection at a distance *x* from the support; $F_{z}$ and $M_{y}$ are the shear forces and bending moments, respectively, acting on the element of the beam; ρ is the density of the structural material; A=wh is the beam cross section; and q(x,t) is a general distributed load per unit length. Merging the two equations yields:

(2) $$\frac{d^{2}M_{y}}{dx^{2}}+q\left(x,t\right)=\rho A\frac{\partial^{2}W\left(x,t\right)}{\partial t^{2}}.$$

Upon the bending of the beam, the length of the neutral plane is given by l=Rdθ, with R and dθ the curvature radius and span angle of the bent beam, respectively. The strain at the planes above and below the neutral plane is given by $\varepsilon_{x}=\frac{\delta l}{l}=\frac{\left(R+z\right)d\theta -Rd\theta }{Rd\theta }=\frac{z}{R}$. Given that the material is elastic, the stress in the *x*-direction can then be calculated as $\sigma_{x}=E\varepsilon_{x}=E\frac{z}{R}$, with *E* indicating the Young’s modulus of the material [2].

The bending moment around the *y*-axis, $M_{y}$, created by the tension forces in *x*-direction, $dF_{x}=\sigma_{x}dA=E\frac{z}{R}dydz$, applied to a distance *z* from the neutral plane, is given by:

(3) $$M_{y}=\int^{\;}dM_{y}=\int^{\;}-z\vec{e_{z}}\times dF_{x}\vec{e_{x}}=\int^{\;}-z^{2}\frac{E}{R}dydz=-\frac{E}{R}I_{z},$$

where $I_{z}=\int_{-h/2}^{h/2}\int_{-w/2}^{w/2}z^{2}dydz$ is the second moment of area of the cross section (for standard rectangular microcantilevers $I_{z}=\frac{wh^{3}}{12}$). Finally, for small curvatures the radius of the curvature can be approximated by $\frac{1}{R}=\frac{\partial^{2}W\left(x,t\right)}{\partial x^{2}}$ [2], and therefore the bending moment can be expressed as:

(4) $$M_{y}=-EI_{z}\frac{\partial^{2}W\left(x,t\right)}{\partial x^{2}}.$$

Substituting Equation (4) into Equation (2) results in a differential equation usually known as the Euler–Bernoulli beam equation [1,2,3]:

(5) $$EI_{z}\frac{\partial^{4}W\left(x,t\right)}{\partial x^{4}}+\rho A\frac{\partial^{2}W\left(x,t\right)}{\partial t^{2}}=q\left(x,t\right).$$

A general solution of this equation is obtained by performing a modal decomposition—i.e., by considering the microcantilever response as the linear superposition of simple vibrational modes. The first step in this process is to extract the natural resonance frequency and shape of each vibrational mode, assuming zero external forces—i.e., q(x,t)=0.

It is assumed that the deflection at any point of the beam varies harmonically with time, so the general solution for each mode can be separated into a temporal term, ψ(t), and a spatial term, Φ(x). The ansatz for the temporal term is a harmonic oscillation with natural angular frequency $\omega_{0,n}$, where the index *n* describes the resonant mode, such as $\psi_{n}\left(t\right)=e^{i\omega_{0,n}t}$. Therefore, the general solution of each individual mode can be written as [4]:

(6) $$W_{n}\left(x,t\right)=\psi_{n}\left(t\right)\Phi_{n}\left(x\right).$$

Inserting the solution of Equation (6) into Equation (5) and rearranging the terms gives:

(7) $$\frac{d^{4}\Phi_{n}\left(x\right)}{dx^{4}}=\beta_{n}^{4}\Phi_{n}\left(x\right),$$

with $\beta_{n}^{4}=\left(\frac{\rho A\omega_{0,n}{}^{2}}{EI_{z}}\right)$. The solution to Equation (7) provides the spatial term of Equation (6):

(8) $$\Phi_{n}\left(x\right)=c_{1}\cos\left(\beta_{n}x\right)+c_{2}\sin\left(\beta_{n}x\right)+c_{3}\cosh\left(\beta_{n}x\right)+c_{4}\;\sin h\left(\beta_{n}x\right).$$

Different boundary conditions are associated with each type of end constraints of the microresonator. For the particular case of the suspended cantilever considered in this paper, the boundary conditions reflect the fact that the displacement, Φ(x), and slope, $\frac{d\Phi \left(x\right)}{dx}$, must be zero at the clamped end (x=0), while the bending moment, $M_{y}$, and shear force, $F_{z}$, are zero at the free-end (x=L) [1,2,3,4]—i.e.:

(9a) Φ(0)=0,

(9b) $$\mathrm{and}\;\frac{d\Phi \left(0\right)}{dx}=0,$$

(9c) $$M_{y}=EI_{z}\frac{\partial^{2}\Phi \left(L\right)}{\partial x^{2}}=0,$$

(9d) $$\mathrm{and}\;F_{z}=EI_{z}\frac{\partial^{3}\Phi \left(L\right)}{\partial x^{3}}=0.$$

Imposing these four boundary conditions on Equation (8) results in a homogeneous system of four equations. Nontrivial solutions are obtained when the determinant of the matrix of the coefficients of these equations vanishes, which corresponds to the condition:

(10) $$\cos\left(\beta_{n}L\right)\;\cos h\left(\beta_{n}L\right)+1=0.$$

**Figure 2:**
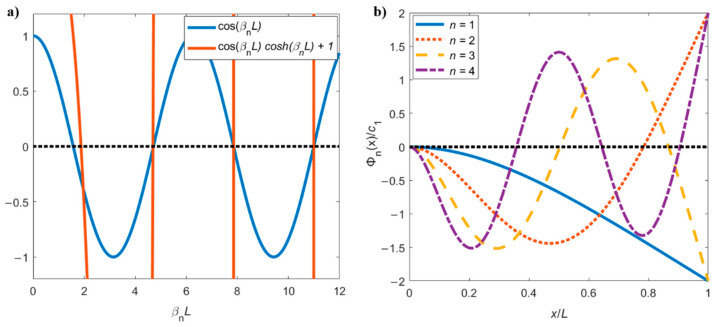
(**a**) Plot of Equation (10) (orange line), whose first four solutions $\beta_{n}L$ (indicated in the text) are the crossings with zero. The blue line shows the asymptotic approximation $\cos\left(\beta_{n}L\right)=0$, which can be solved analytically, and that agrees with the numerical solution for n≥2; (**b**) mode shapes of the first four flexural modes of a cantilever.

Equation (10) can be numerically solved and the first consecutive roots $\left(\beta_{n}L\right)$ calculated, as shown in Figure 2a. Indicatively, $\left(\beta_{1}L\right)=1.875,\;\left(\beta_{2}L\right)=4.694,\;\left(\beta_{3}L\right)=7.855$, $\left(\beta_{4}L\right)=10.996,\;$etc. The condition expressed in Equation (10) can be asymptotically approximated by $\cos\left(\beta_{n}L\right)=0$, with solutions given by $\beta_{n}L=\left(n-\frac{1}{2}\right)\pi$, with n=1, 2, 3, 4,… also shown in Figure 2a [2,4].

The system of equations obtained from imposing the boundary conditions (Equation (9a)–(9d)) in Equation (8) is solved for the constants $c_{1,2,3,4}$. It is found that $c_{1}=-c_{3}$, $c_{2}=-c_{4},$ and $\frac{c_{2}}{c_{1}}=\frac{\sin\left(\beta_{n}L\right)-\sin h\left(\beta_{n}L\right)}{\cos\left(\beta_{n}L\right)+\cos h\left(\beta_{n}L\right)}$. For each particular value of $\beta_{n}L$, the spatial solution, referred to as the flexural mode shape, is obtained by substituting these results into Equation (8) [4]:

(11) $$\Phi_{n}\left(x\right)=c_{1}\left(\cos\left(\beta_{n}x\right)-\cosh\left(\beta_{n}x\right)+\frac{\sin\left(\beta_{n}L\right)-\sin h\left(\beta_{n}L\right)}{\cos\left(\beta_{n}L\right)+\cos h\left(\beta_{n}L\right)}\left(\sin\left(\beta_{n}x\right)-\;\sin h\left(\beta_{n}x\right)\right)\right),$$

where $c_{1}$ remains undefined until an external force is applied. These modal shapes are shown in Figure 2b for a generic $c_{1}$. Note how the number of nodes and the slope near the base of the cantilever increase with the mode number *n*.

The natural angular resonance frequency of each mode, $\omega_{0,n}$, can then be calculated using the respective $\beta_{n}^{4}$, thus obtaining:

(12) $$\omega_{0,n}=\beta_{n}{}^{2}\sqrt{\frac{EI_{z}}{\rho A}}=\frac{{\left(\beta_{n}L\right)}^{2}h}{L^{2}}\sqrt{\frac{E}{12\rho }}.$$

Finally, the complete solution of Equation (6) is [4]:

(13) $$W_{n}\left(x,t\right)=a_{1}e^{i\omega_{0,n}t}\left(\cos\left(\beta_{n}x\right)-\cosh\left(\beta_{n}x\right)+\frac{\sin\left(\beta_{n}L\right)-\sin h\left(\beta_{n}L\right)}{\cos\left(\beta_{n}L\right)+\cos h\left(\beta_{n}L\right)}\left(\sin\left(\beta_{n}x\right)-\;\sin h\left(\beta_{n}x\right)\right)\right).$$

The Euler–Bernoulli beam model only accounts for free vibrations of undamped cantilevers. The free vibrations and mode shapes of clamped-clamped or supported beams can also be predicted using the Euler–Bernoulli model by replacing Equation (9) with the appropriate boundary conditions [1,4]. In addition, more complex continuous beam equations have been developed, which include the effects of rotary inertia and shear deformation [1] or the effect of axial tensile or compressive forces [1,2]. However, these models are seldomly used in sensing applications and therefore fall outside the scope of this review.

### 2.2. Harmonic Oscillations with a Single Degree of Freedom

Although a microcantilever is a continuous system with infinite degrees of freedom, its dynamical response can be accurately described by a single degree of freedom, given that in most applications a specific resonant mode dominates. In fact, these simplified models allow us to extract some useful information from the vibrations, such as the amplitude response and energy dissipation mechanisms, beyond what is provided by the more complex Euler–Bernoulli model. This section presents the theoretical background of the harmonic oscillator models.

#### 2.2.1. Simple Harmonic Oscillator

The simplest possible model for describing the oscillatory motion of the cantilever is the undamped free oscillator. In this case, it is assumed that the cantilever is represented by an effective mass, $m_{\mathrm{eff}}$, connected to a linear elastic spring with stiffness $k_{\mathrm{eff}}$, whose potential energy is given by $U\left(z\right)=\frac{1}{2}k_{\mathrm{eff}}z^{2}$, where *z* is the displacement from the equilibrium position *z* = 0. By using the restitutive force of this spring, $F=-\frac{dU\left(z\right)}{dz}=-k_{\mathrm{eff}}z$ within Newton’s law $F=m_{\mathrm{eff}}\ddot{z}\left(t\right)$, one gets the following equation of motion [3,5]:

(14) $$m_{\mathrm{eff}}\ddot{z}\left(t\right)+k_{\mathrm{eff}}\;z\left(t\right)=0,$$

where the dots represent the time derivative. The solution of this second-order differential equation has the form of an oscillatory motion, described by $z\left(t\right)=A_{0}e^{i\left(\omega t+\phi \right)}$, with $A_{0}$ and ϕ being the amplitude and phase of the motion, respectively, and ω being its frequency. Substituting this solution into Equation (14), one gets the expression for the (angular) natural resonance frequency (for each mode):

(15) $$\omega_{0}=\sqrt{\frac{k_{\mathrm{eff}}}{m_{\mathrm{eff}}}}.$$

This is the natural resonance frequency of the cantilever when it vibrates freely in vacuum, and it is equivalent to the resonance frequency calculated using the Euler–Bernoulli beam theory presented in Equation (12). A classical result from structural mechanics is that the effective stiffness of the cantilever is given by $k_{\mathrm{eff}}=\frac{Ewh^{3}}{4L^{3}}$ [2]. This indicates that each resonant flexural mode predicted by the Euler–Bernoulli model can be described by an equivalent harmonic oscillator with a different $m_{\mathrm{eff}}$. Therefore, by comparing Equation (15) with Equation (12), one can obtain the equivalent mass of each resonant mode in the limit of small damping (cantilevers vibrating in vacuum or air, for example):

(16) $$m_{\mathrm{eff},n}=\frac{3\rho hbL}{{\left(\beta_{n}L\right)}^{4}}=\frac{3m_{c}}{{\left(\beta_{n}L\right)}^{4}},$$

where $m_{c}=\rho Lhw$ is the total mass of the cantilever. Considering, for example, the fundamental resonance mode, where $\beta_{1}L=1.875$ (see Figure 2a), one gets $m_{\mathrm{eff},1}=0.24m_{c}$, as confirmed elsewhere [6,7].

#### 2.2.2. Forced Damped Harmonic Oscillator

By introducing an intrinsic damping coefficient c and an external driving force $F_{drive}\left(t\right)$ in Equation (14), the equation of motion becomes [3,5]:

(17) $$m_{\mathrm{eff}}\ddot{z}\left(t\right)+c\dot{z}\left(t\right)+k_{\mathrm{eff}}z\left(t\right)=F_{drive}\left(t\right).$$

Using Equation (15), defining a parameter $\gamma =\frac{c}{m_{\mathrm{eff}}}=\frac{\omega_{0}}{Q}$, where $Q=\frac{m_{\mathrm{eff}}\;\omega_{0}}{c}$ is called the quality factor and depends on the damping of the system, and assuming a periodic force $F_{drive}\left(t\right)=F_{0}e^{i\omega t}$, Equation (17) is written as:

(18) $$\ddot{z}\left(t\right)+\gamma \dot{z}\left(t\right)+\omega_{0}^{2}z\left(t\right)=\frac{F_{0}}{m_{\mathrm{eff}}}e^{i\omega t}.$$

A steady-state harmonic solution is given by $z\left(t\right)=A_{0}e^{i\left(\omega t+\phi \right)}$, with $A_{0}$ being the amplitude of the motion and ϕ being the phase between the applied external force and the induced motion. Substituting such ansatz into Equation (18) yields:

(19) $$\left(-\omega^{2}+\omega_{0}{}^{2}+i\gamma \omega \right)=\frac{F_{0}}{A_{0}m_{\mathrm{eff}}}e^{-i\phi }.$$

After separating the real and imaginary parts, the following system is obtained:

(20) $$\{\begin{array}{c} \omega_{0}{}^{2}-\omega^{2}=\frac{F_{0}}{A_{0}m_{\mathrm{eff}}}\cos\left(\phi \right)\;\left(a\right)\\ -\gamma \omega =\frac{F_{0}}{A_{0}m_{\mathrm{eff}}}\sin\left(\phi \right)\;\left(b\right) \end{array},$$

which can be solved to get:

(21a) $$A_{0}=\frac{F_{0}/m_{\mathrm{eff}}}{\sqrt{{\left(\omega_{0}{}^{2}-\omega^{2}\right)}^{2}+{\left(\frac{\omega_{0}\omega }{Q}\right)}^{2}}},$$

(21b) $$\phi =\mathrm{atan}\left(-\frac{\omega_{0}\omega }{Q\left(\omega_{0}{}^{2}-\omega^{2}\right)}\right).$$

**Figure 3:**
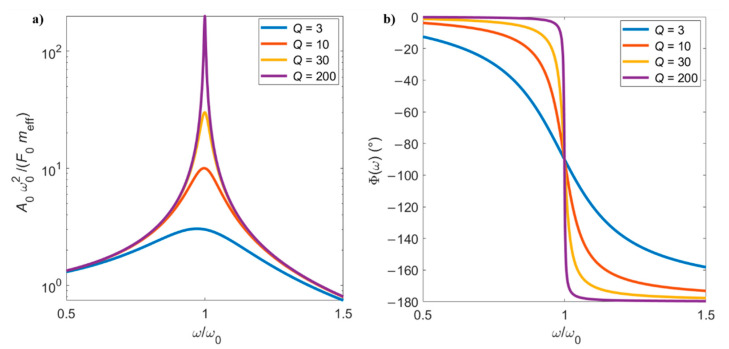
(**a**) Amplitude and (**b**) phase responses as function of the normalized excitation frequency of a forced and damped harmonic oscillator. Four different levels of damping (*Q*) are considered, typically encountered in microcantilevers vibrating in air or liquid mediums.

Equation (21a) gives the amplitude response of the cantilever, whereas Equation (21b) represents the phase between the driving force and the motion of the cantilever. The amplitude and phase curves of microcantilevers with different values of *Q* are shown in Figure 3.

If the intrinsic damping coefficient c tends to zero, then the quality factor Q tends to infinite. In this case, the second term under the square root in Equation (21a) is negligible, and the maximum of the amplitude response will occur at the natural frequency—that is, at $\omega =\omega_{0}$. Conversely, when c increases, Q decreases and the amplitude response of the beam shifts to the left, with the peak of maximum amplitude occurring at a frequency lower than $\omega_{0}$. The maximum amplitude can be obtained by solving $\frac{dA_{0}}{d\omega }=0$, which gives the resonance frequency of intrinsically damped vibrations.

(22) $$\omega_{res}=\omega_{0}\sqrt{1-\frac{1}{2Q^{2}}}.$$

As observed in Figure 3, low *Q*s decrease the peak amplitude, broadening the peak and shifting the maximum of the curve towards lower values. An even more pronounced shift occurs when the cantilever oscillates in damped environments, such as in the case of liquids, as will be discussed in the next section. The motion of the microcantilever is in phase with the drive force before the resonance, but lags 90° behind at the resonance point, and 180° after resonance. Low values of *Q* result in a wider linear region of phase change around the natural frequency. The phase of the resonator is of particular importance when closed-loop excitation systems are used, as will be discussed in Section 3.

The physical definition of *Q* is the ratio of the energies stored, $E^{\prime }$, and dissipated, $\Delta E^{\prime \prime }$, in the resonator, both averaged per oscillation cycle, at the resonance frequency [5], $Q=2\pi \frac{E^{\prime }}{\Delta E^{\prime \prime }}$. *Q* can be assessed experimentally from the amplitude response of the resonator using the bandwidth method by the expression:

(23) $$Q\approx \frac{\omega_{res}}{\Delta \omega_{-3dB}}=\frac{\omega_{res}}{{\left(\omega_{2}-\omega_{1}\right)}_{-3dB}},$$

where $\omega_{res}$ corresponds to the maximum of the amplitude curve (Equation (22)), and $\Delta \omega_{-3dB}$ is the bandwidth at 3 dB below the maximum amplitude. In practice, measuring $\Delta \omega_{-3dB}$ corresponds to determining the frequencies of the two points, $\omega_{2}$ and $\omega_{1}$, at half the power (3 dB or 70.7%) of the maximum of the amplitude response.

#### 2.2.3. General One-Degree-of-Freedom Equation of Motion for Microcantilevers

Despite the usefulness of the simple models discussed above, these are still of limited applicability to describe the complete dynamical response of a microcantilever in most applications. In fact, for sensing applications very often additional terms must be considered according to the excitation strategy used, the type of samples to be tested, or the properties of the surrounding fluid. A more general one-degree-of-freedom equation of motion can then be introduced as:

(24) $$\ddot{z}+\frac{\omega_{0}}{Q}\dot{z}+\omega_{0}^{2}\left[1+\lambda \cos\left(\Omega t+\Phi \right)\right]z+\frac{\alpha }{m_{\mathrm{eff}}}z^{3}+\frac{\eta }{m_{\mathrm{eff}}}z^{2}\dot{z}+\frac{\delta }{m_{\mathrm{eff}}}\left(z{\dot{z}}^{2}+z^{2}\ddot{z}\right)\;=\;\frac{1}{m_{\mathrm{eff}}}\left[F_{drive}\left(t\right)+F_{interaction}\left(z,\dot{z},\ddot{z},\ldots \right)\right]\;,$$

where z represents the displacement of the microcantilever, $m_{\mathrm{eff}}$ is the effective mass, α is the nonlinear spring constant (also known as the Duffing parameter [8,9]), η is the coefficient of nonlinear damping, δ is the coefficient of nonlinear inertia [10,11], $F_{drive}\left(t\right)$ is a general external drive signal, $F_{interaction}\left(z,\dot{z},\ddot{z},\ldots \right)$ is a general interaction force between the cantilever and the surrounding environment, and λcos(Ωt+Φ) is a function (proportional to the displacement of the beam) that is used to modulate the spring constant at frequency Ω, phase Φ (with respect to the drive force) and gain λ. Equation (24) is capable of describing distinct sources of non-linearities and different types of possible excitation mechanisms and external forces, and is applicable to any vibrational mode by using the respective effective mass and natural resonance frequency. This general equation can be used to describe rich and complex dynamics of the vibrating beam [4,12,13,14,15,16,17] across a wide variety of sensing applications, as will be discussed in more detail in the following sections.

### 2.3. Operation in Dissipative Fluids

To theoretically describe a cantilever beam moving in a fluid, Equation (5) is used and the general distributed load q(x,t) is substituted with a driving force to excite the motion of the beam and a hydrodynamic load induced by the fluid [18]:

(25) $$EI_{z}\frac{\partial^{4}W\left(x,t\right)}{\partial x^{4}}+c_{0}\frac{\partial W\left(x,t\right)}{\partial t}+m_{0}\frac{\partial^{2}W\left(x,t\right)}{\partial t^{2}}=F_{drive}\left(x,t\right)+F_{hydro}\left(x,t\right).$$

Compared to Equation (5), the dissipative intrinsic viscous term per unit length, $c_{0}=c/L$, was also introduced and $m_{0}=\rho A=\rho wh$ is the mass of the cantilever per unit length.

Usually, the hydrodynamic load is decomposed into two terms: a pressure drag (inertial term, proportional to the acceleration) and a viscous drag (dissipative term, proportional to the velocity) [18]—i.e.,

(26) $$F_{hydro}\left(x,t\right)=-m_{A}\frac{\partial^{2}W\left(x,t\right)}{\partial t^{2}}-c_{V}\frac{\partial W\left(x,t\right)}{\partial t},$$

where $m_{A}$ is an added mass and $c_{V}$ an added damping coefficient, both expressed per unit length. Equation (25) can then be re-written as:

(27) $$EI_{z}\frac{\partial^{4}W\left(x,t\right)}{\partial x^{4}}+\left(c_{0}+c_{V}\right)\frac{\partial W\left(x,t\right)}{\partial t}+\left(m_{0}+m_{A}\right)\frac{\partial^{2}W\left(x,t\right)}{\partial t^{2}}=F_{drive}\left(x,t\right).$$

In [19], Sader solved Equation (27) analytically and showed that, in the limit of small dissipative effects (i.e., for $Q_{n}\gg 1$), the resonance frequency of the extrinsically damped vibrations in fluid with added mass and added damping ($m_{A}$ and $c_{V}$), $\omega_{R,n}$, and the respective quality factor, $Q_{n}$, of each mode of the cantilever are given by [20]:

(28a) $$\frac{\omega_{R,n}}{\omega_{res,n}}={\left(1+\frac{m_{A}}{m_{0}}\right)}^{-\frac{1}{2}},$$

(28b) $$Q_{n}=\frac{\omega_{R,n}\;\left(m_{0}+m_{A}\right)}{\left(c_{0}+c_{V}\right)},$$

where $\omega_{res,n}$ is the resonance frequency of each mode for intrinsically damped vibrations, calculated by Equation (22). Furthermore, Sader also showed that the amplitude response of each mode is given by [19]:

(29) $$X≅{\left({\left(\omega_{R,n}{}^{2}-\omega^{2}\right)}^{2}+{\left(\frac{\omega_{R,n}\omega }{Q_{n}}\right)}^{2}\right)}^{-1/2},$$

with X being a normalized amplitude. Equation (29) is readily identified as the amplitude response of the forced damped harmonic oscillator, as given by Equation (21a).

In summary, provided the dissipation is low; $Q_{n}\gg 1$; and, therefore, the resonant modes do not overlap, each resonant mode of a cantilever vibrating in liquid can be described by the harmonic oscillator model, with a resonance frequency $\omega_{R,n}$ and a quality factor $Q_{n}$.

The added mass, $m_{A}$, and added viscous damping coefficient, $c_{V}$, can be analytically calculated as functions of the properties of the liquid. This is done by solving the incompressible Navier–Stokes equations [21] and determining the velocity and pressure fields in the moving fluid that create a hydrodynamic load, Γ(ω), acting on the oscillating beam (see [22,23] for cantilevers with circular and rectangular cross sections, respectively).

The hydrodynamic load $\Gamma \left(\omega \right)=\;\Gamma^{\prime }\left(\omega \right)+i\;\Gamma^{\prime \prime }\left(\omega \right)$ is a dimensionless function that contains inertial (real part, added mass) and dissipative (imaginary part, viscous damping) terms. For example, the added mass and viscous damping acting on a rectangular cantilever are described by [19,20]:

(30a) $$m_{A}=\frac{\pi }{4}\rho_{f}w^{2}\Gamma_{rect}{}^{\prime }\left(\omega \right),$$

(30b) $$c_{V}=\frac{\pi }{4}\rho_{f}w^{2}\omega \Gamma_{rect}{}^{\prime \prime }\left(\omega \right),$$

where $\Gamma_{rect}{}^{\prime }\left(\omega \right)$ and $\Gamma_{rect}{}^{\prime \prime }\left(\omega \right)$ are the real and imaginary parts of the hydrodynamic load acting on a cantilever with rectangular cross section, $\Gamma_{rect}\left(\omega \right)$, which is a function of the Reynolds number $Re=\frac{\rho_{f}\omega w^{2}}{4\eta }$, with $\rho_{f}$ and η being the density and viscosity of the fluid, respectively.

Since it is not trivial to calculate $\Gamma_{rect}\left(\omega \right)$ analytically (see [23] for details), Sader used the strategy of correcting the hydrodynamic load calculated by Chen for a cylindrical cantilever, $\Gamma_{circ}\left(\omega \right)$ [22], with an empirical function Ω(ω), in the form of $\Gamma_{rect}\left(\omega \right)=\Omega \left(\omega \right)\Gamma_{circ}\left(\omega \right)$, to match the analytical results for $\Gamma_{rect}\left(\omega \right)$ of Tuck [23]. Although treatable, Sader’s correction function is still lengthy and numerical [19].

In a subsequent work, Maali et al. [20] further simplified the problem, by using simple polynomials to fit the real and imaginary parts of Sader’s solution for $\Gamma_{rect}\left(\omega \right)$, thus obtaining the analytical expressions:

(31a) $$\Gamma_{rect}{}^{\prime }\left(\omega \right)=a_{1}+a_{2}\frac{\delta }{w}=a_{1}+\frac{a_{2}}{w}\sqrt{\frac{2\eta }{\rho_{f}\omega }},$$

(31b) $$\Gamma_{rect}{}^{\prime \prime }\left(\omega \right)=b_{1}\frac{\delta }{w}+b_{2}{\left(\frac{\delta }{w}\right)}^{2}=\frac{b_{1}}{w}\sqrt{\frac{2\eta }{\rho_{f}\omega }}+\frac{2\eta }{\rho_{f}\omega }{\left(\frac{b_{2}}{w}\right)}^{2},$$

where $\delta =\sqrt{\frac{2\eta }{\rho_{f}\omega }}\;$is the thickness of the layer surrounding the cantilever in which the velocity of the fluid drops by a factor of 1/*e* and *a*_1_ = 1.0553, *a*_2_ = 3.7997, *b*_1_ = 3.8018, and *b*_2_ = 2.7364. These expressions are valid for Reynolds numbers ranging between 1 and 1000 [20], which are the typical values for most of the microcantilever-based sensing applications, and can be applied for the first resonance modes. Equations (28)–(31) can be used in conjunction to obtain the dependence between the resonance frequency and quality factor of the resonant mode *n* and the rheological properties of the fluid, as will be shown in Section 5.

The results discussed in this section for the flexural oscillations of a microcantilever immersed in viscous fluids were experimentally validated in [24] and then extended to model torsional vibrations of the cantilever in [25], which can also be used in applications such as atomic force microscopy (AFM). The effect of a nearby wall on the frequency response of flexural and torsional oscillations of a cantilever immersed in viscous fluids was studied in [26]. Exact analytical solutions of the three-dimensional flow field and hydrodynamical load were presented, for the general case of a thin-blade (cantilever) performing flexural and torsional oscillations in viscous fluids, by Van Eysden and Sader [27]. These analytical calculations for the three-dimensional hydrodynamical load were subsequently incorporated in the initial analysis of flexural [19] and torsional [25] oscillations and used for the general case of arbitrary mode orders [28].

## 3. Excitation Schemes and Noise

### 3.1. Excitation Strategies

Dynamic sensing applications, especially when operating fluids, require an external force to induce vibrations of measurable amplitude and acceptable levels of signal-to-noise ratio. To maximize the efficiency of excitation, an external force must be applied at different frequencies corresponding to the microcantilevers resonant modes. This actuation is typically accomplished by using electrical, thermal, or acoustic actuation techniques [29]. Regardless of the physical mechanism used to generate the driving force ($F_{drive}$ in Equation (24)), the excitation strategy can be broadly divided into two families, according to the way $F_{drive}$ is applied and tuned. These two methodologies, namely “external excitation” and “feedback excitation” are briefly reviewed below.

#### 3.1.1. External or Open-Loop Excitation Mechanisms

Traditional dynamic sensing applications are based on the assumption that the cantilever response can be well approximated by a single degree of freedom harmonic oscillator, as shown in Section 2.2.2. The adsorption of analytes on the sensor surface (for mass sensing application) and/or changes in the properties of the surrounding fluid (for rheology sensors) induce a shift in the natural frequency $\omega_{0}$ and, potentially, a change in the quality factor *Q* as well. Such changes can be experimentally measured by performing frequency sweeps of the excitation force applied to the microcantilever and recording the corresponding amplitude response at each frequency, ideally recovering the theoretical curves shown in Figure 3. Such excitation mode is denoted as “open-loop” or “external”, as the force applied to the cantilever is entirely controlled by the user, without feeding back any information from the cantilever response.

**Figure 4:**
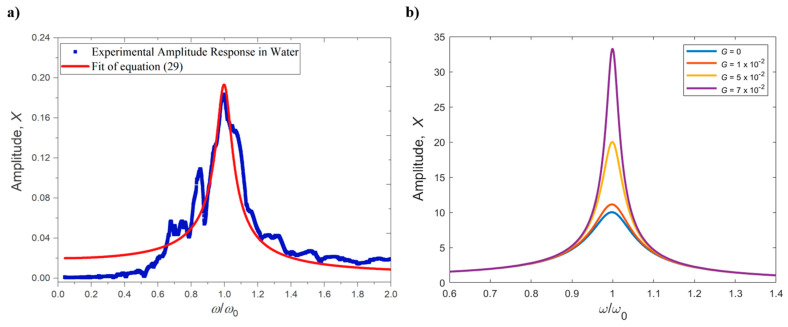
(**a**) Experimental amplitude response of an acoustically excited microcantilever oscillating in water, showing a noisy peak not accurately fitted by the theoretical amplitude curve; (**b**) dependence of the amplitude response of a microcantilever on the gain of the feedback loop in the *Q*-control method, for $\omega_{0}=1$ rad/s, original Q=10 (for *G* = 0), $\frac{F_{0}}{m_{\mathrm{eff}}}=1$ N/kg and $\phi =\frac{\pi }{2}$ rad.

Most commonly, the excitation force is generated by a piezoelectric actuator embedded in the cantilever support which can set it into oscillation by creating an acoustic wave that propagates through the substrate materials. When the frequency of such wave matches (or is reasonably close to) the resonance frequency of the cantilever, the oscillation amplitude can be significant and detectable for measurements (as described in Section 2). This setup is the most commonly used for imaging and sensing in air or a vacuum, due to its simplicity and ease of operation. However, it presents major drawbacks for sensing applications in dissipative fluids. Indeed, most of the dynamic sensing applications are based on detecting small changes in the resonance frequency of the probe (see Section 4 and Section 5), measured by performing frequency sweeps with the excitation force and detecting the maximum amplitude of the cantilever deflection. However, the forces acting on the cantilever by the surrounding fluid dramatically increase the amount of dissipation, as explained in Section 2.3, and therefore the *Q* factor of the resonant response is low (around 10 or below). As shown in Figure 3, in this condition the peak in the amplitude response is not very pronounced, making it difficult to: (i) tune the frequency of the excitation force to match the resonance and (ii) detect small changes in the resonance frequency itself induced by changes in the properties of the fluid. Even worse, a typical response of an acoustically excited cantilever suffers from the well-known “forest of peaks” phenomenon, where several spurious peaks appear in the amplitude response due to mode coupling with oscillations in the fluid and in the sample holder [30]. In these cases, identifying the right “peak”—corresponding to the cantilever resonance—and measuring small variations in its position becomes very challenging, as shown in Figure 4a for the case of a microcantilever oscillating in water and externally excited with a piezoelectric holder. The proper choice of material to be used as mechanical holder is key to obtaining reduced spurious peaks in the amplitude and phase curves [31].

To partially overcome these drawbacks, alternative physical sources have been considered to generate the driving force, with the aim of moving the point of excitation closer to the cantilever free end and minimizing mode coupling with the surrounding fluid. A common proposed technique is magnetic excitation [32], but nowadays thermal excitation, via an additional laser shining on the microcantilever [33] or by thermal effects in bi-layer cantilevers [34], and piezoelectric [35] or electrostatic [36] excitations are also commonly used for exciting the microdevices. With the point of application of the exciting force being co-located with the detection point (typically the cantilever tip), there is no need for a travelling wave to be formed in the probe and therefore the delay/phase-shift between excitation and deflection is minimized and there is very limited coupling with fluid vibratory modes. This results in a measured amplitude spectrum that is much closer to the theoretical one shown in Figure 3.

However, irrespective of the experimental implementation details of open-loop excitation, the fundamental problem of low *Q* factor and, correlated to this, a relatively inaccurate measurement of the frequency of the resonance peak remains.

#### 3.1.2. Feedback or Closed-Loop Excitation Mechanisms

An alternative approach to improve the sensitivity and signal-to-noise ratio of the cantilever when used as a mass or rheology sensor is based on acting on the dynamic response of the cantilever itself, instead of on the physical actuation and detection methodology. Theoretically, if the *Q* factor of the amplitude response is significantly increased, then the issues related to poor signal-to-noise ratio in the identification of the resonance peak and the “forest of peaks” phenomenon become less pronounced. To achieve this goal, “feedback” or “closed-loop” approaches are needed, where the excitation force depends on the response of the microcantilever itself. Within this umbrella, several techniques have been proposed in the literature.

The original idea was proposed by Rodríguez et al. [37] and is commonly known as “*Q*-control”, where the measured microcantilever deflection is amplified by a gain, delayed by an user-defined quantity and then added to the external harmonic excitation with a feedback loop. In its basic form, the equivalent single degree of freedom dynamics reads:

(32) $$\ddot{z}+\frac{\omega_{0}}{Q}\dot{z}+\omega_{0}^{2}z=\frac{1}{m_{\mathrm{eff}}}\left[F_{0}\cos\omega t-k_{\mathrm{eff}}Gz\left(t-\frac{\phi }{\omega }\right)\right].$$

When compared to Equation (24), $F_{drive}=F_{0}\cos\omega t-k_{\mathrm{eff}}Gz\left(t-\frac{\phi }{\omega }\right)$ consists of a harmonic external excitation with amplitude $F_{0}$ and frequency ω (same as in open-loop strategies), added up to a forcing component obtained by delaying the instantaneous detected deflection of the microcantilever z(t) by a time $\tau_{delay}=\frac{\phi }{\omega }$ (with ϕ being a user-defined phase shift along the feedback loop) and then amplifying the delayed deflection by a feedback *Q*-control gain, $k_{\mathrm{eff}}G$. It can be shown that by modulating the feedback gain *G*, the effective *Q* factor exhibited by the cantilever response is much larger than the intrinsic *Q* factor of the cantilever, as shown in Figure 4b [37].

An alternative and more effective approach, based on the concept of parametric resonance, has been proposed by Moreno et al. and Prakash et al. [38,39,40]. In these works, Equation (24) turns into:

(33) $$\ddot{z}+\frac{\omega_{0}}{Q}\dot{z}+\omega_{0}^{2}\left[1-G\cos\left(2\omega_{0}t\right)\right]z+\frac{\alpha }{m_{\mathrm{eff}}}z^{3}=\frac{F_{tip-sample}}{m_{\mathrm{eff}}},$$

where the cantilever is parametrically excited ($F_{drive}\left(t\right)=0$) by a signal proportional to the displacement of the cantilever, amplified by *G* and at twice the natural frequency of the beam, $2\omega_{0}$, while the non-linear force acting on the tip of the cantilever, $F_{interaction}=F_{tip-sample}$, was used for imaging [41]. Once again, changes in the feedback gain *G* translate to variation in the effective *Q* factor, as shown in [14].

Similarly, Miller et al. [42] studied the possibility of using the nonlinear damping coefficient η to parametrically control the phase of a nonlinear resonator and implement a parametric phase-locked loop (PLL), turning Equation (24) into:

(34) $$\ddot{z}+\frac{\omega_{0}}{Q}\dot{z}+\omega_{0}^{2}\left[1+\lambda \cos\left(2\left(\omega_{0}t+\Phi \right)+\delta \right)\right]z+\frac{\alpha }{m_{\mathrm{eff}}}z^{3}+\frac{\eta }{m_{\mathrm{eff}}}z^{2}\dot{z}=0,$$

where the parameter δ in Equation (34) is the phase setpoint of the PLL. Again, this technique does not require any external force $F_{drive}$, as the probe is self-oscillating.

It is worth noting that all the techniques discussed so far are capable of dramatically improving the cantilever response, but the feedback gains always have upper bounds corresponding to the probe oscillation becoming unstable and (theoretically) growing exponentially.

A different approach has been proposed by the authors in [43], where the intrinsic dynamics of the cantilever is first made unstable by amplifying the deflection signal and then stabilized by introducing a nonlinear saturation in the feedback loop. The resulting dynamics reads:

(35) $$\ddot{z}+\frac{\omega_{0}}{Q}\dot{z}+\omega_{0}^{2}z=\frac{1}{m_{\mathrm{eff}}}\left[\mathrm{sat}\left(Kz\left(t-\tau_{delay}\right)\right)-m_{A}\ddot{z}-c_{A}\dot{z}\right].$$

In this work, the cantilever is subjected to a fluidic force given by $F_{interaction}=F_{fluidic}\left(\dot{z},\ddot{z}\right)=-m_{A}\ddot{z}-c_{A}\dot{z}$, where $m_{A}$ and $c_{A}$ describe the added mass and damping induced by the fluid. Additionally, the drive force of the cantilever has the form $F_{drive}\left(t\right)=\mathrm{sat}\left(Kz\left(t-\tau_{delay}\right)\right)$, where the deflection *z* of the microcantilever is delayed by $\tau_{delay}$ (microseconds) along the feedback loop and amplified by a gain *K*, before being saturated and fed back as the driving force.

Although analytical solutions of Equation (35) are not available, accurate predictions of the overall response can be obtained by using Harmonic Balance methods [44]. These techniques are described in detail in [44,45], and it is assumed that the deflection in Equation (35) is approximately harmonic—i.e.:

(36) $$z\left(t\right)≅A_{0}\sin\left(\omega t\right).$$

If this solution is inserted into Equation (35), the response of the saturation can be expanded in harmonic terms (an approach known as Describing Function, see [44,45] for details) and all the terms of the same frequency are balanced together, resulting in analytical values for the critical gain *K* that initiates the self-oscillations, and amplitude and frequency of oscillation. According to classical Nyquist theory [44], the saturation term is required to stabilize the self-oscillations of the feedback loop for non-zero $\tau_{delay}$. However, no upper bound exists for *K*, with the self-sustained oscillation remaining always stable, and therefore this excitation technique requires less tuning effort when compared with the other strategies described previously. This method was used to study a microcantilever used for imaging [46], for mass sensing [47], and for rheology sensor [48].

### 3.2. Detection Mechanisms

The mechanisms that can be employed for measuring the deflection and resonance share some of the physical mechanisms used for actuation, and include optical, capacitive, piezoelectric and piezoresistive sensing. Arguably the most common method to study the vibration of microstructures are the optical lever technique, where the motion of the cantilevers can be detected by reflecting a laser from the beam into a position-sensitive detector, and laser Doppler vibrometry, an interference-based optical technique. The two former methods depend on external equipment that is difficult to miniaturize or integrate, reducing the range of applications. However, this also stimulates an on-going effort to develop alternative optical detection methods to reduce the complexity of the detection configuration [49], using off-the-shelf components (DVD optical pickups, for example [50]), or implementing more recent paradigms, such as quantum sensing [51]. In the piezoresistive technique, the cantilever is fabricated with an integrated resistor having piezoresistive properties, and therefore the electrical resistance changes as a function of cantilever motion [52]. The use of piezoresistive detection is advantageous compared to optical detection because there is no need for laser or detector alignments. A disadvantage is that the current flowing in the piezoresistive layer causes temperature fluctuations in the cantilever, which may lead to parasitic cantilever deflection and to piezoresistive changes [53]. Electrostatic (capacitive) detection is also a widespread approach, which is possible to integrate with compact electronics. The capacitive detection is due to the changes in capacitance that arise from the displacement of the resonator relative to one or more fixed electrodes/gates (the resonator and the electrode/s are separated by a small gap, forming a capacitor). The capacitance method provides a high sensitivity and absolute displacement. However, the capacitance method is troublesome in an electrolyte solution due to the Faradic current between the resonator and electrodes, which obscures the desired signal. Another disadvantage is that capacitive sensing loses efficiency for nanoscale devices: capacitance scales as area/separation but practical limits on resonator–gate gaps limit reduction of their dimensions, and given the higher resonance frequencies of NEMS compared with MEMS, a large fraction of the drive and detection signals are lost through parasitic capacitances [54].

### 3.3. Noise

Many cantilever-based applications require detecting frequency shifts caused by small changes at the sensor surface or in the surrounding medium, as discussed in Section 4 and Section 5 for the cases of mass and rheology sensing, respectively. Any real case resonator or oscillator shows fluctuations in its amplitude and frequency/phase responses, caused by the noise present in the mechanical system, surrounding environment and equipment, resulting in uncertainties in the measurements. Therefore, understanding how noise affects such measurements is key to assess the ultimate sensing performance. This section discusses how to experimentally measure noise, its physical origins, and how to use it to estimate the minimum detectable frequency shift and corresponding limit of detection.

#### 3.3.1. Time Domain—Allan deviation

It is possible to quantify the frequency stability of an oscillator or resonator system in the time domain using the Allan variance (a measure of the frequency drift in a specific time window). The Allan deviation,$\;\sigma_{y}\left(\tau \right)$*,* is defined as the root mean square of the differences between consecutive relative frequency measurements taken in non-overlapping time windows of duration *τ* [55,56,57]:

(37) $$\sigma_{y}\left(\tau \right)=\sqrt{\frac{1}{2\left(M-1\right)}\sum_{i=1}^{M-1}{\left(\frac{f_{i+1}-f_{i}}{f_{c}}\right)}^{2}},$$

where *M* is the total number of frequency measurements taken, $f_{i}$ is the *i*th frequency measurement (averaged in the time window with duration τ), and $f_{c}$ is the nominal carrier frequency (typically the resonance frequency of the microcantilever, $f_{0}$). The Allan deviation depends on the time interval used to collect consecutive samples, commonly denoted as τ, or the integration time.

For short integration times, the Allan variance is dominated by the frequency/phase noise in the resonator and associated circuit. For long integration times, frequency drifts due to temperature variations, resonator ageing and other environmental conditions dominate. More information on the physical origin and manifestation of the noise mechanisms in MEMS/NEMS resonators can be found in [56,57,58,59] and will be shortly discussed later.

#### 3.3.2. Frequency Domain—Spectral Densities

Another metric to study the frequency stability of the system is the noise density around the carrier (resonance) frequency. For continuous signals over time, such as the vibrations of a microresonator or oscillator, power spectral densities (PSD) can be defined. These show how the power of a signal is distributed over the frequency spectrum and are hence customarily called power spectra. For convenience, and to be able to apply the concept to any kind of signals (not only physical power), spectral densities are also defined by the variance of the signal over time or, in other words, by the squared deviation of the signal from its mean value over time. The variance of the signal at a certain frequency is then interpreted as the energy of the signal at that frequency (measured using a well-defined bandwidth in each frequency point).

Therefore, considering the frequency domain, frequency/phase instabilities can be measured by the spectral density of normalized frequency/phase fluctuations. These are given by:

(38a) $$S_{y}\left(f\right)=y_{rms}^{2}\left(f\right)\frac{1}{BW},$$

(38b) $$S_{\Phi }\left(f\right)=\Phi_{rms}^{2}\left(f\right)\frac{1}{BW},$$

where $S_{y}\left(f\right)$ and $S_{\Phi }\left(f\right)$ are, respectively, the spectral density of frequency and phase fluctuations; $y_{rms}\left(f\right)$ and $\Phi_{rms}\left(f\right)$ are the measured root mean squared (rms) value of normalized frequency and phase, respectively, in a band of Fourier frequencies containing the carrier frequency $f_{c}$; and BW is the width of the frequency band in Hz. The units of $S_{y}\left(f\right)$ are 1/Hz and of $S_{\Phi }\left(f\right)$ are rad^2^/Hz. $S_{y}\left(f\right)$ and $S_{\Phi }\left(f\right)$ are one-sided spectral densities, and apply over a Fourier (or sideband) frequency range *f* from 0 to ∞ [60,61]. The relation between these two quantities is given by [61]:

(39) $$S_{\Phi }\left(f\right)={\left(\frac{f_{c}}{f}\right)}^{2}S_{y}\left(f\right).$$

$S_{\Phi }\left(f\right)$ has been historically utilized in metrology, but more recently the single-sideband phase noise, ℒ(f), has become the prevailing quantity to measure phase noise among manufacturers and users of frequency standards [60]. ℒ(f) is the noise power density normalized to the carrier power and can be defined as the ratio [60]:

(40) $$ℒ\left(f\right)=\frac{P_{noise\;\left(1\;Hz\right)}\left(f\right)}{P_{signal\;}},$$

where $P_{noise\;\left(1\;Hz\right)}\left(f\right)$ (units of dBm/ Hz) is the power density in one single sideband due to phase modulation (PM) by noise (for a 1 Hz bandwidth), $\mathrm{and}\;P_{signal\;}$(units of dBm) is the power of the carrier. Usually, ℒ(f) is expressed in decibels as $\log_{10}ℒ\left(f\right)$ and its units are dBc/Hz (dB below the carrier, where the power in each point was measured considering a 1 Hz bandwidth). Devices shall be characterized by a plot of ℒ(f) versus Fourier (or offset) frequency *f*, as shown in [62], for example. The fact that Equation (40) is approximately half of $S_{\Phi }\left(f\right)$ (for small mean squared phase deviation [60]), led to a recent redefinition of ℒ(f) as:

(41) $$ℒ\left(f\right)=\frac{1}{2}S_{\Phi }\left(f\right).$$

#### 3.3.3. Conversion between Frequency and Time Domain—Power Law Spectral Densities

It has been shown, from both theoretical considerations and experimental measurements, that spectral densities due to random noise can be accurately modeled by power-laws, where the spectral densities vary as a power of the Fourier frequency *f* [63]. More specifically, $S_{y}\left(f\right)$ and $S_{\Phi }\left(f\right)$ can be written as the sums:

(42a) $$S_{y}\left(f\right)=\sum_{\alpha =-2}^{+2}h_{\alpha }f^{\alpha },\;0<f<f_{h},$$

(42b) $$S_{\Phi }\left(f\right)=\sum_{\alpha =-2}^{+2}f_{c}^{2}h_{\alpha }f^{\alpha -2},\;0<f<f_{h},$$

with $f_{h}$ as the high-frequency cut-off of an infinitely sharp low-pass filter (frequency after which the $S_{\Phi }\left(f\right)$ spectrum becomes flat) and $h_{\alpha }$ as the constants associated with each type of noise process. The two equations are related by Equation (39).

**Figure 5:**
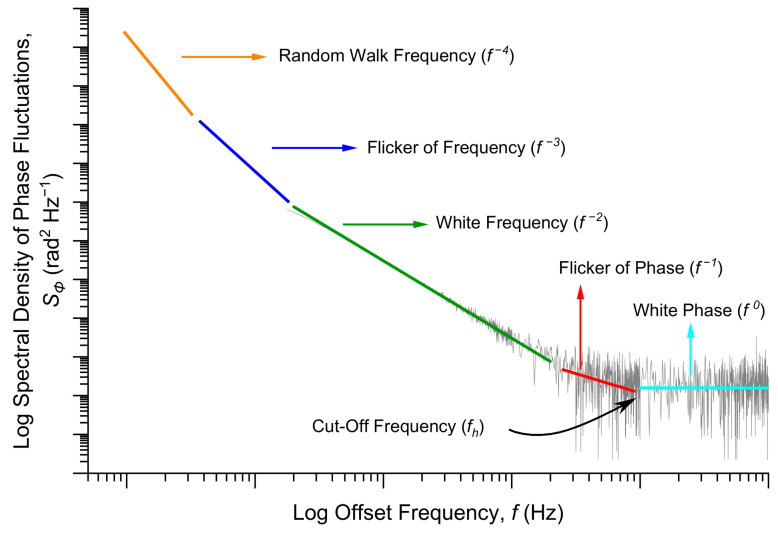
Power-laws of the spectral density of phase fluctuations, $S_{\Phi }\left(f\right)$, and corresponding noise mechanisms. A typical experimental curve of $S_{\Phi }\left(f\right)$ is plotted in light grey. The cut-off frequency (*f_h_*) is also indicated.

The random fluctuations are often represented by the sum of five independent noise processes: random walk frequency noise (constant $h_{-2}$, $f^{-4}$ dependence on Fourier frequency), flicker of frequency ($h_{-1}$,$\;f^{-3}$ dependence on frequency), white frequency noise ($h_{0}$, $f^{-2}$ dependence on frequency), flicker of phase ($h_{1}$, $f^{-1}$ dependence on frequency), and white phase noise ($h_{2}$ and $f^{0}$ dependence on frequency), as can be seen in Figure 5.

Cutler and Searle derived the calculation of the Allan deviation, $\sigma_{y}\left(\tau \right)$, from the spectral density of the frequency fluctuations, $S_{y}\left(f\right)$, using the formula [60,63,64]:

(43) σy(τ)=[∫0∞Sy(f)|H(f)|2df]12=[2∫0fhSy(f)sin4(πτf)(πτf)2df]12,

where ${\left|H\left(f\right)\right|}^{2}=2\frac{\sin^{4}\left(\pi \tau f\right)}{{\left(\pi \tau f\right)}^{2}}$ is the transfer function of an infinitely sharp low-pass filter, with $2\pi f_{h}\tau \gg 1$, used to count and average the frequency values for a time τ [60,63].

This integral can be analytically (for simple cases) or numerically solved as shown in [56,65,66,67] using the expressions for $S_{y}\left(f\right)$ given by Equation (42a). For the case of Equation (43), one obtains:

(44) $$\sigma_{y}\left(\tau \right)=\sqrt{h_{-2}A\tau^{1}+h_{-1}B\tau^{0}+h_{0}C\tau^{-1}+h_{1}D\tau^{-2}+h_{2}E\tau^{-2}},$$

with $A=\frac{2\pi^{2}}{3}$, B=2ln(2), $C=\frac{1}{2}$, $D=\frac{1.038+3\ln\left(2\pi f_{h}\tau \right)}{4\pi^{2}},$ and $E=\frac{3f_{h}}{4\pi^{2}}$. Different dependencies of the Allan deviation with τ can be found. Additionally, the results depend on the type of filter chosen (other types of filters, such as a single-pole filter, can be used).

Experimentally, the slopes observed in the $S_{\Phi }\left(f\right)$ (or the equivalent ℒ(f)) spectrum can be fitted to power laws of the form $y=ax^{b}$, from where the constants $h_{\alpha }$ are extracted and the Allan deviation $\sigma_{y}\left(\tau \right)$ for a certain integration time τ is computed.

For micromechanical resonators in an oscillator configuration, the Allan deviation can be measured directly by probing the frequency of the signal at the oscillator output. For this purpose, an appropriate frequency counter can be used. Alternatively, the phase noise of the MEMS oscillator can be measured by using a high-stability reference oscillator and a phase detector, which can provide the instantaneous phase difference between the reference and the MEMS oscillator under test. Alternatively, a spectrum analyzer can be used to measure the single-sideband phase noise, provided that the resolution bandwidth of the instrument is narrower than the width of the resonance peak of the MEMS resonator. Furthermore, the reference oscillator for such measurements must exhibit significantly lower phase noise than the device under test. However, when using a sweeping spectrum analyzer, the amplitude noise and the phase noise of the cantilever oscillator are convoluted. If the amplitude noise of the device under test (MEMS oscillator or resonator) is comparable to its phase noise, then other techniques involving phase detectors, PLLs or delay lines must be employed to isolate and measure the phase noise (these can be found in many signal source analyzers).

#### 3.3.4. Physical Origins of Noise

Readers are referred to the works [57,58,59,68] for a thorough and detailed analysis of some of the intrinsic noise mechanics present in MEMS resonators. In these works, analytical expressions for the phase noise (frequency domain) and Allan deviation (time domain) are derived for the cases of thermomechanical fluctuations, temperature fluctuations, adsorption-desorption events, defect motion and moment exchange in gaseous environments [58,59,68]. In [56], the authors show an extensive experimental work to study the frequency fluctuations induced by the experimental setup and also by nonlinear phenomena, such as nonlinear damping, nonlinear mechanical properties (Duffing parameter) or nonlinear mode coupling in the resonators. Interestingly, they conclude that the frequency fluctuations that have been consistently observed within the MEMS community have no known physical origin, yet, or that current practices must be rethought.

As shown in [68], the Allan deviation can be calculated for various functional forms of the phase noise density. For example, considering a frequency noise with a 1/*f* component, the frequency fluctuations are given by $S_{y}\left(f\right)=Z\left(\frac{f_{c}}{f}\right)$ or, equivalently, $S_{\Phi }\left(f\right)=Z{\left(\frac{f_{c}}{f}\right)}^{3}$, where Z is a scale factor which encloses physical information about the noise process. Solving Equation (43) for the $S_{y}\left(f\right)$ of 1/*f* noise, one obtains an Allan deviation given by:

(45a) $$\sigma_{y}\left(\tau \right)=\sqrt{2\ln\left(4Z\pi f_{c}\right)\tau^{0}}.$$

Note that the Allan deviation caused by the 1/*f* frequency noise mechanism is independent of the integration time τ. Additionally, the constant $h_{-1}$ (shown in Equation (44)) can be related with the physical parameters of this type of noise. Another example from [68] is related to frequency drift, in which the frequency fluctuations are given by $S_{y}\left(f\right)=Z{\left(\frac{f_{c}}{f}\right)}^{2}$ or, equivalently, $S_{\Phi }\left(f\right)=Z{\left(\frac{f_{c}}{f}\right)}^{4}$, and the Allan deviation becomes:

(45b) $$\sigma_{y}\left(\tau \right)=\sqrt{\frac{2\pi^{2}}{3}Zf_{c}\tau^{1}},$$

being now dependent on $\tau^{1}$, with Z related to $h_{-2}$ (see Equation (44)). Finally, for white frequency noise fluctuations, $S_{y}\left(f\right)=Z$ or, equivalently, $S_{\Phi }\left(f\right)=Z{\left(\frac{f_{c}}{f}\right)}^{2}$, the Allan deviation becomes:

(45c) $$\sigma_{y}\left(\tau \right)=\sqrt{\pi Z\tau^{-1}},$$

dependent on $\tau^{-1}$ and with Z related to $h_{0}$ (see Equation (44)).

To conclude this section, an expression derived in [59,68,69] for the Allan deviation caused by events of adsorption-desorption of residual molecules around the resonator is discussed. The Allan deviation caused the adsorption-desorption events, $\sigma_{y_{AD}}\left(\tau \right)$, is given by:

(46) $$\sigma_{y_{AD}}\left(\tau \right)=\sqrt{\frac{N_{a}\tau_{r}\sigma_{occ}{}^{2}}{2}\tau^{-1}}\frac{\delta m_{AD}}{m_{c}},$$

where $\delta m_{AD}$ is the adsorbed-desorbed molecule mass, $m_{c}$ is the total mass of the resonator, $\sigma_{occ}$ is the variance in the occupation probability of any given site on the surface, $N_{a}$ is the Avogadro number and $\tau_{r}$ is the correlation time for an adsorption-desorption event, with τ the integration time. This equation can be important for mass sensing applications and will be used later in this review, in Section 4.3.

#### 3.3.5. Minimum Detectable Frequency Shift, ${\delta f}_{\min}$

A key question for any frequency-based sensing application is: what is the minimum measurable frequency shift, $\delta f_{min}$, that can be resolved in a realistic noisy system? As suggested in [58], in principle $\delta f_{min}$ should be a shift comparable to the mean square noise in an average of series of frequency measurements, or in other words:

(47) $$\delta f_{min}\approx \frac{1}{N}\sqrt{\sum_{i=1}^{N}{\left(f_{i}-f_{0}\right)}^{2}},$$

where $f_{i}$ represents the consecutive *i*th frequency measurements and $f_{0}$ is the natural resonance frequency of a particular resonant mode vibrating in a dissipative medium.

Alternatively, to determine this minimum frequency shift, $\delta f_{min}$, one can also resort to the definition of the Allan deviation $\sigma_{y}\left(\tau \right)\;$measured for a given integration time *τ* (Equation (37)), and think of:

(48) $$\delta f_{min}\approx \sigma_{y}\left(\tau \right)f_{0},$$

where $\sigma_{y}\left(\tau \right)$ is the Allan deviation caused by all the sources of noise in the system. In the next section, we show how this value of the minimum detectable frequency shift can be used to determine the ultimate limits of detection of the resonating sensors.

## 4. Mass Sensing

MEMS devices microfabricated with a wide variety of geometries and materials have been extensively used as mass sensors with different operating conditions. The minimum mass resolution ever reported in the literature is 1.7 yg (the mass of a proton), achieved by using a carbon nanotube resonator and demanding experimental conditions, such as a low-noise measurement setup, high-vacuum and cryogenic temperatures [70]. Nanoelectromechanical (NEM) resonators working in high-vacuum conditions have also been used as mass spectroscopes, to detect single biological molecules that adsorb in real time on the surface of the resonators, one by one, several hundred times [71,72,73]. Despite these remarkable demonstrations of mass sensing in vacuum, in some applications the cantilever must be able to operate in dissipative media, such as air and liquid, since these fluids are often the substance to be tested or the support for the analytes to be detected.

**Figure 6:**
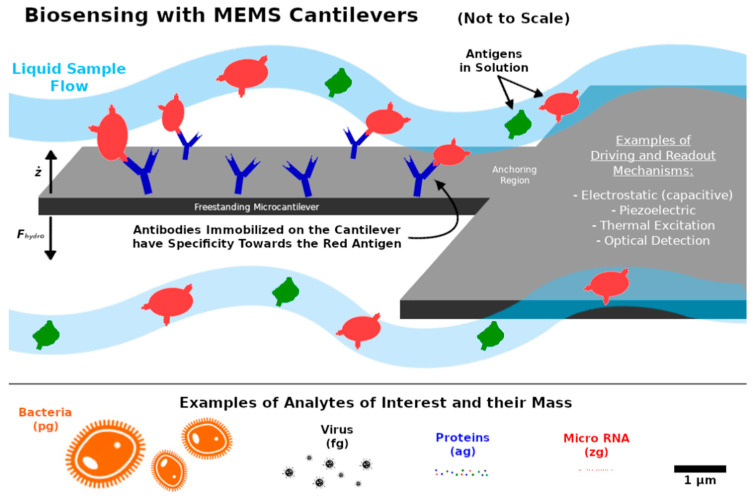
Illustration of a biosensing strategy with a microcantilever. The cantilever is functionalized with antibodies on the top surface (these are the biorecognition agents, which are chemically bound to the surface). Due to the specific binding of analytes (in this simple example, the red antigens are captured by the antibodies but the green ones are not), a resonance frequency shift or a static deflection will occur. On the bottom, the scale of the size and mass of bacteria, virus particles, proteins, and micro RNA strands is illustrated.

In particular, microcantilevers have been used to detect single virus, organic vapors, prostrate-specific antigen, and other analytes of interest in very small concentrations [74,75,76,77,78]. For some mass sensing applications, such as in the case of biosensing, the cantilever is typically functionalized with molecules that act as receptors and have a high affinity and selectivity for the analyte to be detected, as illustrated in Figure 6. The strategy used to immobilize the molecular probes on the surface of the microcantilever depends on the chemical nature of both the molecules and the surface. Depending on the target analyte to be detected, one can use antibodies, synthetic oligonucleotides, locked nucleic acids (LNA), or aptamers as recognition elements [79]. An illustrative comparison between the scale of common biological entities and MEMS sensors is also given in Figure 6.

Some advantages of using microcantilevers as mass sensors include the possibility of probing the microscale, a high sensitivity due to their small dimensions, the ability to detect several analytes simultaneously, the fact that the detection does not require the analytes to be tagged (label-free detection), and the relative simplicity in interfacing the sensor with electronic readout and microfluidics [80].

### 4.1. Dynamic vs Static Sensing Modes

**Figure 7:**
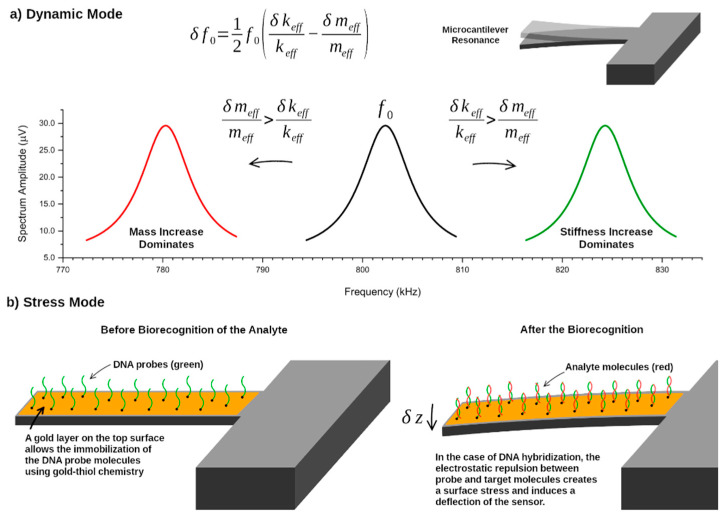
Mass sensing strategies. (**a**) Dynamic mode: the mass adsorbed on the surface of the cantilever causes changes in both its effective mass and stiffness, resulting in a shift in the resonance frequency, $\delta f_{0}$, to lower or higher values, depending on which effect dominates (red and green spectra). (**b**) Static deflection mode: the sensor deflects by a quantity δz due to intermolecular interactions at the surface of the sensor. In the case of a DNA biosensing experiment, the surface stress is due to the electrostatic repulsion between the molecules at the surface when complementary DNA (red strands) hybridizes with the initially immobilized DNA probes (green strands).

Two distinct mechanisms can be used for mass sensing with a microcantilever. In the first, called dynamic mode, the microcantilever oscillates with a constant frequency (as discussed in Section 2) and the binding of the target analyte induces a shift in this frequency (caused by changes in the effective mass and/or stiffness of the cantilever), which can be detected. In the second method, called static deflection mode, the cantilever is initially static and bends when the target analyte binds to the surface. This deflection, which can be detected, is caused by surface stress arising from the electrostatic repulsion between the molecules at the surface, or due to steric hindrance, for example. These two mass sensing modes are illustrated in Figure 7. In this work, only the dynamical mass sensing mode will be discussed. For a review of the static deflection mode, the reader is referred to [81,82,83].

### 4.2. Mass Sensitivity

The dependence of the resonance frequency of the cantilever operating in vacuum with its effective mass and stiffness is obtained by differentiating Equation (15) with respect to these parameters, $\delta f_{0}=\frac{\delta f_{0}}{\delta k_{\mathrm{eff}}}\delta k_{\mathrm{eff}}+\frac{\delta f_{0}}{\delta m_{\mathrm{eff}}}\delta m_{\mathrm{eff}}$. The final result is given by [84]:

(49) $$\delta f_{0}=\frac{1}{2}f_{0}\left(\frac{\delta k_{\mathrm{eff}}}{k_{\mathrm{eff}}}-\frac{\delta m_{\mathrm{eff}}}{m_{\mathrm{eff}}}\right),$$

where $k_{\mathrm{eff}}$ and $m_{\mathrm{eff}}$ are the effective stiffness and mass of the resonant mode, respectively, and $\delta k_{\mathrm{eff}}$ and $\delta m_{\mathrm{eff}}$ are the corresponding infinitesimal changes. This equation can be applied to any flexural mode by using the appropriate effective mass (see Equation (16)). Equation (49) indicates that the resonance frequency$\;f_{0}$ of a mass sensor shifts when its effective mass and/or stiffness change due to the adsorption of an analyte on its surface. As discussed for the case of static mass sensing, the presence of analytes on the surface of the cantilever results in a surface stress. This surface stress also affects the resonance frequency of the cantilever and, therefore, the sensor behavior in the dynamical sensing mode. Several attempts have been made to relate the presence of surface stresses with changes in the effective stiffness of the cantilever [85,86], but a complete understanding of the physical mechanisms still remains elusive. This, in turn, impedes us from completely decoupling the simultaneous effects of the added mass and the surface stress (which impacts the effective stiffness) on the resonance frequencies [87].

However, when the adsorption of mass results in a distributed and low-density layer on the cantilever surface, or when the mass is adsorbed near its free end, the mass change ($\delta m_{\mathrm{eff}}$) effect dominates. Conversely, a high-density mass layer, or mass adsorbed near the support end of the microcantilever, causes a dominant stiffness-change effect ($\delta k_{\mathrm{eff}}$) [88,89]. These two competing effects are illustrated in Figure 7 and can be simultaneously measured by using two distinct vibrational modes of the resonator, provided that the adsorption position of the analyte is known [90]. In general, multimode excitation can be used to extract information about the mass, stiffness, and/or position of the analyte by measuring the resonance frequency shifts induced by the analyte on several flexural modes [91,92].

The mass sensitivity of a sensor operating in vacuum ($S_{mass,vac}$) is defined as the frequency shift induced by a mass added to the resonators (units of Hz kg^-1^) and can be evaluated from Equation (49) as:

(50) $$S_{mass,vac}=\frac{\delta f_{0}}{\delta m_{\mathrm{eff}}}=-\frac{f_{0}}{2m_{\mathrm{eff}}},$$

where the stiffness change has been assumed to be negligible ($\delta k_{\mathrm{eff}}\;\sim \;0$). This equation shows that small cantilever effective mass and high natural resonance frequency are advantageous features to achieve high mass sensitivities in a vacuum.

To estimate the mass sensitivity for microcantilevers vibrating in fluids, one can at first combine Equations (15), (22), and (28a), thus expressing the resonance frequency with added mass and added damping, $\omega_{R,n}$, as:

(51) $$\omega_{R,n}=\omega_{res,n}{\left(1+\frac{m_{A}}{m_{0}}\right)}^{-\frac{1}{2}}={\left(\frac{k_{\mathrm{eff}}}{m_{\mathrm{eff},n}}\right)}^{\frac{1}{2}}{\left(1-\frac{1}{2Q^{2}}\right)}^{\frac{1}{2}}{\left(1+\frac{Lm_{A}}{\beta_{n}^{\prime }m_{\mathrm{eff},n}}\right)}^{-\frac{1}{2}},$$

where, as defined previously, $\omega_{res,n}$ is the resonance frequency of the *n*^th^ mode of the intrinsically damped resonator, $m_{A}$ and $m_{0}$ are the added mass by the fluid and the mass of the resonator, both per unit length, with $m_{c}=Lm_{0}=\frac{m_{\mathrm{eff},n}{\left(\beta_{n}L\right)}^{4}}{3}=\beta_{n}^{\prime }m_{\mathrm{eff},n}$, as shown in Equation (16).

The mass sensitivity in fluid, $S_{mass,fluid}$, for the *n*^th^ mode, can then be obtained from Equation (51), by differentiating it with respect to these several parameters, $\delta f_{R}=\frac{\delta f_{R}}{\delta k_{\mathrm{eff}}}\delta k_{\mathrm{eff}}+\frac{\delta f_{R}}{\delta Q}\delta Q+\frac{\delta f_{R}}{\delta Lm_{A}}\delta Lm_{A}+\frac{\delta f_{R}}{\delta m_{\mathrm{eff}}}\delta m_{\mathrm{eff}}$. After some cumbersome calculations (see [93] for details) and admitting that the variations in the quality factor (defined in Equation (28b)) and stiffness are negligible ($\delta k_{\mathrm{eff}}\;\sim \;0$ and δQ ~ 0), one obtains:

(52) $$\delta f_{R}=-\frac{f_{R}}{2}\left(\frac{L}{m_{c}+Lm_{A}}\right)\delta m_{A}-\frac{f_{R}}{2}\left(\frac{\beta_{n}^{\prime }}{m_{c}+Lm_{A}}\right)\delta m_{\mathrm{eff}}.$$

Finally, assuming that the properties of the fluid do not change, and therefore $\delta m_{A}\;\sim \;0$ (see Equations (30a) and (31a) and Section 5 for cases where they do change), the mass sensitivity in fluids is given by:

(53) $$S_{mass,fluid}=\frac{\delta f_{R}}{\delta m_{\mathrm{eff}}}=-\frac{f_{R}}{2}\left(\frac{\beta_{n}^{\prime }}{m_{c}+Lm_{A}}\right).$$

This expression indicates that a higher mass sensitivity in fluid is obtained with microcantilevers with reduced dimensions (eventually nanometric), which have small total mass $m_{c}$ and high resonance frequency $f_{R}$, and for higher resonance modes (big values of $\beta_{n}^{\prime }=\frac{{\left(\beta_{n}L\right)}^{4}}{3}$). Additionally, a smaller added mass $m_{A}$ from the hydrodynamic load (see Equations (30) and (31)) is advantageous.

### 4.3. Limits of Detection (LoD)

The minimum mass that a resonator can detect is called limit of detection (*LoD*) and is defined as the mass of analyte that causes a frequency shift equal to three times the minimum detectable frequency shift $\delta f_{min}$ [55], given a general sensitivity S:

(54) $$LoD=3\frac{\delta f_{min}}{S}.$$

As seen in Equations (47) and (48), $\delta f_{min}$ depends on the stability of the resonance frequency, which, in its turn depends on the noise present in the system (Allan deviation). On the other hand, the mass sensitivity (*S*) depends on the operating conditions and can take the value of $S_{mass,\;vac}$ or $S_{mass,fluid}$ (Equations (50) and (53)).

In many applications, the sensor cannot operate under vacuum conditions. As seen, the operation of dynamic-mode mass sensors in gas or liquid media is challenging because the oscillation is damped by the fluid, decreasing *S* while increasing $\delta f_{min}$, and both factors contribute to the degradation of the mass *LoD*. The consequence is that MEMS flexural-mode resonators have typical limits of detection of hundreds of pg in air and even higher in liquids, rendering them ineffective for the detection of small analytes at low concentrations directly in liquid media.

In Section 3, the Allan deviation and phase noise were discussed in detail, and these formulas and methods allow the correct and complete characterization of the frequency stability of cantilever resonators and, in particular, mass sensors, as this stability has a critical effect on the *LoD*. However, in most of the mass sensing demonstrations found in literature, noise and/or frequency stability are very rarely mentioned. Instead, some different metrics, such as the quality factors or standard deviation, are arbitrarily used [94,95,96,97,98,99,100,101,102,103,104,105,106,107,108,109], making it difficult to compare the *LoD*s achieved in the different works. Works that show a detailed characterization of the frequency/phase stability can be found in [62,110,111,112,113,114,115,116]. Establishing coherent standards to be used when developing or assessing cantilever-based mass sensor should be encouraged [117].

To conclude this section, let us consider Equation (46) of Section 3 and combine it with Equation (50) (neglecting changes in stiffness). If it is assumed that: (i) in Equation (50), the shift in frequency caused by the adsorption-desorption events is the minimum detectable frequency shift ($\delta f_{0}\;\sim \;\delta f_{min}$); (ii) the adsorbed-desorbed mass in Equation (46) contributes entirely for the change in effective mass of the cantilever in Equation (50), $\delta m_{AD}\sim \;\delta m_{\mathrm{eff}}$; and (iii) $m_{c}=\beta_{n}^{\prime }m_{\mathrm{eff}}$ (Equation (16)), one gets for $\delta f_{min}$:

(55) $$\delta f_{min}=\sigma_{y_{AD}}\left(\tau \right)f_{0}Y\beta_{n}^{\prime },$$

with Y=(2Naτrσocc2τ−1)−12 as a numerical constant. This is a similar expression to that obtained in [113], and shows that the $\delta f_{min}$ and consequently the *LoD* caused by mass adsorption-desorption events depend on the Allan deviation, the integration time, the natural frequency of the resonator, the resonance mode, and the physical constants in a non-trivial way.

As seen in Equation (54), the limit of detection can be decreased by increasing the sensitivity of the devices and/or reducing the minimum detectable frequency shift. However, as confirmed by Equations (53) and (55), both these parameters can have very complex dependences with geometry, vibration mode, physical processes of mass transfer or even the added mass by the surrounding dissipative fluid. This last term gains even more relevance when flexural vibration modes are used, since these modes displace a lot of fluid. This effect can be greatly reduced by using torsional or extensional modes in a mass sensor, for example, but also explored advantageously for rheological studies and the extraction of the fluid properties, as further discussed in Section 5. Flexural cantilevers can be useful for obtaining mass and rheology measurements simultaneously due to the close interaction between the resonator and the medium.

## 5. Viscosity Sensing

The vibrating microcantilever can be used for measuring the rheological properties of the fluid it is immersed in. In this section, some strategies to measure the viscosity and density of Newtonian fluids and the dynamic viscosity of non-Newtonian viscoelastic fluids are presented and discussed. The section starts with a brief revision on the response of viscoelastic materials to an applied shear stress.

### 5.1. Viscoelastic Materials

The viscosity, η, of a Newtonian incompressible fluid is defined as the proportionality constant between an applied shear stress to the fluid and the resulting shear strain rate. This is formally described by:

(56) $$\tau_{A}=\eta {\dot{\delta }}_{D},$$

where $\tau_{A}$ is the applied shear stress and $\delta_{D}$ is the shear strain, with ${\dot{\delta }}_{D}=\frac{d\delta_{D}}{dt}$ being the shear strain rate. This equation describes a purely viscous dashpot (hence the index “*D”*), where the shear force is proportional to the velocity. In a Newtonian fluid, the viscosity is constant and does not depend on the shear strain rate. Equation (56) is the simplest possible description of a viscous fluid and can be applied to some common liquids and gases, such as water or air.

However, most of the fluids of interest to biological applications do not follow Newton’s law of viscosity (Equation (56)) and show a much more complex response to an applied shear stress. These fluids are termed non-Newtonian and their viscosity depends on the applied shear rate. A very common response of many solutions is shear-thinning, in which the viscosity decreases with an increasing shear rate. This arbitrarily complex behavior stems from the fact that these fluids have also an elastic response, in addition to the viscous response, and are hence also called viscoelastic fluids.

A Maxwell fluid is the simplest description of a viscoelastic fluid. This model considers that an elastic spring (obeying to Hooke’s law) is added in series with the viscous dashpot, typical of purely viscous materials. The stress–strain relationship in an elastic spring is given by:

(57) $${\dot{\tau }}_{A}=G_{0}{\dot{\delta }}_{S},$$

where ${\dot{\tau }}_{A}$ is the applied shear stress rate, $G_{0}$ is the elasticity constant of the fluid, and $\delta_{S}$ is the shear strain of the spring (index “*S*”) with ${\dot{\delta }}_{S}=\frac{d\delta_{S}}{dt}$ the shear strain rate. The total strain rate of the spring-dashpot series, ${\dot{\delta }}_{tot}={\dot{\delta }}_{D}+{\dot{\delta }}_{S}$, when subjected to a shear stress is given by adding Equations (56) and (57):

(58) $$\tau_{A}+\lambda {\dot{\tau }}_{A}=\eta {\dot{\delta }}_{tot},$$

with $\lambda =\frac{\eta }{G_{0}}$ as a characteristic relaxation time. In the limit $G_{0}\underset{}{\to }\infty ,\dot{\delta_{S}}\underset{}{\to }0$, Equation (58) reduces to Equation (56) and the fluid is purely viscous. In the case of $\eta \underset{}{\to }\infty ,\;\dot{\delta_{D}}\underset{}{\to }0$, Equation (58) reduces to Equation (57) and the fluid is purely elastic. Assuming that the applied shear stress and consequent total strain response are periodic with frequency ω and that the strain response of the material lags behind the applied stress by a phase φ, one gets:

(59a) $$\tau_{A}=\tau_{0}e^{i\left(\omega t+\varphi \right)},$$

(59b) $$\delta_{tot}=\delta_{0}e^{i\omega t},$$

(59c) $$G^{*}=\frac{\tau_{0}}{\delta_{0}}e^{i\varphi },$$

where $G^{*}$ is a dynamic elastic modulus, defined by dividing the applied stress by the total strain of the system, and $\tau_{0}$ and $\delta_{0}$ are the amplitude of the shear stress and total strain, respectively. Equation (59c) reduces, respectively, to Hooke’s and Newton’s laws when the shear stress and strain are in phase (φ=0, $G^{*}=G_{0}$) or in quadrature ($\varphi =\frac{\pi }{2}$, $G^{*}=\eta$). Viscoelasticity corresponds to any other value of φ. By substituting Equations (59a)–(59c) into Equation (58) and rearranging [32]:

(60) $$\tau_{0}e^{i\omega t}\left(1+i\omega \lambda \right)=\delta_{0}\eta i\omega \underset{}{\Rightarrow }G^{*}=\frac{\omega^{2}\lambda^{2}G_{0}}{1+\omega^{2}\lambda^{2}}+i\frac{\omega \lambda G_{0}}{1+\omega^{2}\lambda^{2}}\underset{}{\Rightarrow }G^{*}=G^{\prime }+iG^{\prime \prime },$$

$G^{*}$ is therefore defined as the sum of an elastic part, $G^{\prime }=\frac{\omega^{2}\lambda^{2}G_{0}}{1+\omega^{2}\lambda^{2}}$, and a viscous part, $G^{\prime \prime }=\frac{\omega \lambda G_{0}}{1+\omega^{2}\lambda^{2}}$. The phase lag between the shear stress and the shear strain is given by $\varphi =\arctan\left(\frac{G^{\prime \prime }}{G^{\prime }}\right)$.

The dynamic modulus $G^{*}$ can be used to define a complex dynamic viscosity $\eta^{*}$, by equalling Newton’s and Hooke’s laws through the shear stress applied to the system:

(61) $$\eta^{*}{\dot{\delta }}_{tot}=G^{*}\delta_{tot}\underset{}{\Rightarrow }\eta^{*}=\frac{G^{*}}{i\omega }=\frac{G^{\prime \prime }}{\omega }-i\frac{G^{\prime }}{\omega }\underset{}{\Rightarrow }\eta^{*}=\eta^{\prime }-i\eta^{\prime \prime }.$$

**Figure 8:**
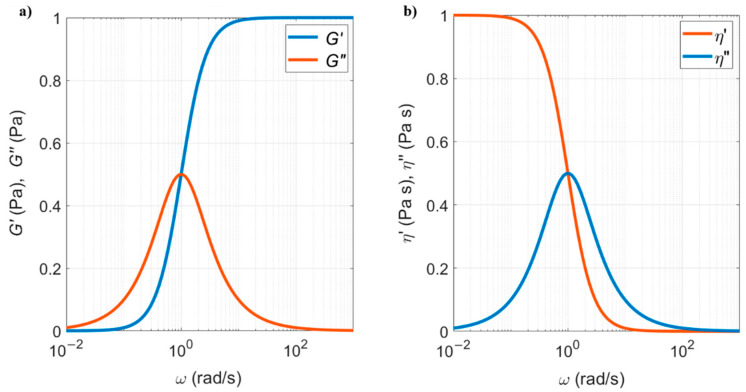
(**a**) Elastic (real) and viscous (imaginary) parts of the dynamic elastic modulus; (**b**) viscous (real) and elastic (imaginary) parts of the complex viscosity for λ=1 s, $G_{0}=1$ Pa, and η=1 Pa s in a Maxwell fluid.

Therefore $\eta^{\prime }=\frac{G^{\prime \prime }}{\omega }=\frac{\lambda G_{0}}{1+\omega^{2}\lambda^{2}}$ is the purely viscous part and $\eta^{\prime \prime }=\frac{G^{\prime }}{\omega }=\frac{\omega \lambda^{2}G_{0}}{1+\omega^{2}\lambda^{2}}$ is the elastic viscosity. The components of both the dynamic modulus and dynamic viscosity are shown in Figure 8, as function of the frequency of the shear load.

At low excitation frequencies, the fluid is purely viscous ($\eta^{\prime }=1$ and $\eta^{\prime \prime }=0$), while at high frequencies it is purely elastic ($G^{\prime }=1$ and $G^{\prime \prime }=0$). When excited at intermediate frequencies, the Maxwell fluid is viscoelastic. The transition between viscous and elastic regimes occurs when the two curves cross each other.

### 5.2. Measuring Rheological Properties of Fluids Using Microcantilevers

#### 5.2.1. Newtonian Fluids

Microcantilevers have been widely used to measure the rheological properties of Newtonian fluids. An early example of such a measurement is provided in [118], where the dependence of the resonance frequency of a cantilever on the viscosity of several aqueous solutions is used to monitor a chemical reaction. This section details how the rheological properties of Newtonian fluids can be measured using the theoretical framework described in Section 2. Two different strategies will be discussed.

The first strategy consists of measuring the amplitude response of the *n*th mode of the vibrating cantilever immersed in a viscus fluid. The experimentally measured amplitude response is fitted to Equation (29), allowing us to simultaneously extract the resonance frequency, $\omega_{R,n},$ and the quality factor, $Q_{n}$. Subsequently, the added inertial mass, $m_{A}$, and the added damping coefficient, $c_{V}$, are extracted from $\omega_{R,n}$ and $Q_{n}$ using Equations (28a) and (28b). Finally, the viscosity, η, and density of the fluid, $\rho_{f}$, can be simultaneously calculated using Maali’s description of the hydrodynamic load by solving the system of equations given by (30) and (31). The sequence can be summarized as follows:

$$\mathrm{fit}\;X≅{\left({\left(\omega_{R,n}{}^{2}-\omega^{2}\right)}^{2}+{\left(\frac{\omega_{R,n}\omega }{Q_{n}}\right)}^{2}\right)}^{-1/2}\underset{}{\Rightarrow }\{\begin{array}{c} \omega_{R,n}\\ Q_{n} \end{array}\underset{}{\Rightarrow }\{\begin{array}{c} m_{A}\\ c_{V} \end{array}\underset{}{\Rightarrow }\{\begin{array}{c} \eta \\ \rho_{f} \end{array}.$$

This strategy has been described in [119,120], for example, and also used in [121] for the longitudinal modes of a microcantilever (not described here) and in [122,123] with a force applied at the free end of the cantilever. An interesting example of a self-excited microcantilever whose phase is used to measure the viscosity of different solutions is shown in [48].

Typically, iterative or numerical methods must be used to solve the system of equations that allow determining η and $\rho_{f}$. Additionally, an initial calibration is required in which the resonance frequency and quality factor of the cantilever vibrating in air ($\omega_{res,n}$) are measured. Furthermore, the fitted Equation (29) only describes the amplitude response around the resonance, and therefore only the rheological properties of the fluid at this frequency are measured.

To overcome this limitation, a second strategy has been developed in [124]. In this method, the amplitude and phase spectra are both experimentally measured for a wide range of excitation frequencies. Then, Equation (19), describing the complete transfer function of the *n*th mode of the forced damped harmonic oscillator oscillating in a fluid with resonance frequency $\omega_{R,n}$ and $\gamma =\frac{\left(c_{0}+c_{V}\right)}{\left(m_{0}+m_{A}\right)}$, is used:

(62) $$\frac{A_{0}m_{\mathrm{eff}}}{F_{0}}e^{i\phi }=\frac{1}{1-{\left(\frac{\omega }{\omega_{R}}\right)}^{2}+\;i\;\frac{\left(c_{0}+c_{V}\right)}{\left(m_{0}+m_{A}\right)}\;\frac{\omega }{\omega_{R}{}^{2}}}.$$

Substituting Sader’s result of $\omega_{R}=\omega_{res}{\left(1+\frac{m_{A}}{m_{0}}\right)}^{-\frac{1}{2}}$ (Equation (28a)), using $e^{i\phi }=\cos\left(\phi \right)+i\sin\left(\phi \right)$, and considering a general ratio of amplitudes $\left|\frac{H\left(\omega \right)}{H_{0}}\right|=\frac{A_{0}m_{\mathrm{eff}}}{F_{0}}$, one gets the following complete transfer function:

(63) $$\left|\frac{H\left(\omega \right)}{H_{0}}\right|\left(\cos\left(\phi \right)+i\sin\left(\phi \right)\right)=\frac{1}{1-{\left(\frac{\omega }{\omega_{res}}\right)}^{2}\left(1+\frac{m_{A}}{m_{0}}\right)+i\;\frac{\left(c_{0}+c_{V}\right)}{m_{0}}\frac{\omega }{\omega_{res}{}^{2}}}.$$

Finally, it is possible to use the experimentally measured amplitude H(ω) and phase ϕ(ω) spectra to determine the added inertial mass and damping coefficient, $m_{A}$ and $c_{V}$, respectively, for all frequencies (not only limited to resonance), by equating the real and imaginary parts of the transfer function as:

(64a) $$\left\{ \begin{array}{c} Re(\frac{H(\omega )}{H_{0}})=|\frac{H(\omega )}{H_{0}}|\cos\left(\phi (\omega )\right)=(1-{\left(\frac{\omega }{\omega_{res}}\right)}^{2}\left(1+\frac{m_{A}}{m_{0}})\right){\left|\frac{H(\omega )}{H_{0}}\right|}^{2}\\ Im(\frac{H(\omega )}{H_{0}})=|\frac{H(\omega )}{H_{0}}|\sin\left(\phi (\omega )\right)=\left(-\frac{(c_{0}+c_{v}}{m_{0}}\frac{\omega }{{\omega_{res}}^{2}}\right){\left|\frac{H(\omega )}{H_{0}}\right|}^{2} \end{array}\right.$$

(64b) $$\left\{ \begin{array}{c} m_{A}=[(1-|\frac{H_{0}}{H(\omega )}|\cos\left(\phi (\omega )\right)){\left(\frac{\omega_{res}}{\omega }\right)}^{2}-1]m_{0}\\ c_{V}=-|\frac{H_{0}}{H(\omega )}|\sin\left(\phi (\omega )\right)\frac{{\omega_{res}}^{2}}{\omega }m_{0}-c_{0} \end{array}\right.$$

The rest of the procedure is identical to the first method: the viscosity, η, and density of the fluid, $\rho_{f}$, are determined from $m_{A}$ and $c_{V}$ using Equations (30) and (31). The fact that the rheological properties of the fluid can be measured for all range of frequencies is crucial for accurate measurements of viscoelastic fluids, as shown in Figure 8, since the properties of a viscoelastic fluid depend on the frequency of the shear load. This will be discussed in the next section.

To determine the limit of detection (*LoD*) of viscosity changes in a Newtonian fluid, Equation (54) can still be used, but where *S* is now the viscosity sensitivity ($S_{viscosity,fluid}$).

To determine the viscosity sensitivity of the microcantilever, the inertial added mass, given by Equations (30a) and (31a), can be differentiated with respect to the fluid viscosity and density, $\delta m_{A}=\frac{\delta m_{A}}{\delta \eta }\delta \eta +\frac{\delta m_{A}}{\delta \rho_{f}}\delta \rho_{f}$, to obtain:

(65) δmA=π4wa2(ρf2ηω)12δη+π4w2a1δρf+π4wa2(η2ρfω)12δρf.

This equation can then be substituted in Equation (52) to calculate the shift in resonance frequency caused by the added mass. Assuming that the variations in fluid density and effective mass of the cantilever are negligible ($\delta \rho_{f\;}\sim \;0$ and $\delta m_{\mathrm{eff}}\;\sim \;0$), one obtains:

(66) δfR=−fR2(Lmc+LmA)(π4wa2(ρf2ηω)12)δη.

By rearranging this equation, substituting $m_{A}$ from Equations (30a) and (31a) and considering the frequency of oscillation at resonance $\omega =2\pi f_{R}$, the viscosity sensitivity in liquids is finally obtained:

(67) Sviscosity,fluid=δfRδη=−fR2[(ρf4πfRη)124mca2πLw+ρfwa1a2+(ηρfπfR)12].

The dependence of the sensitivity with the different geometrical parameters and fluid properties is complex and the design of the microcantilever can be optimized to reach an improved sensitivity and *LoD*. For example, these ideas have been applied by Dufour et al. for the development of a gas sensor in [125].

#### 5.2.2. Viscoelastic Fluids

Measuring the viscoelastic properties of soft matter and fluids has become the focus of extensive research, given the key role these fluids play in biology and food manufacturing, just to cite two examples of relevant applications. Recently, several methods have emerged as powerful tools to investigate the dynamics and structure of soft matter or fluids at the micro or nanoscale [126,127]. One of these methods consists of using the microcantilever in a standard Atomic Force Microscopy (AFM) setup [128] and using the tip-sample interaction to probe the viscoelastic response of live cells [129,130] or soft surfaces [131].

In this section, the use of the microcantilever to measure the rheological properties of a viscoelastic fluid, in the context of the theoretical framework developed in the previous sections, is discussed.

This technique was initially proposed in [132,133], and the main idea is to incorporate the dynamic complex viscosity, $\eta^{*}=\eta^{\prime }-i\eta^{\prime \prime }$, of Equation (61) into the hydrodynamic load (added inertial mass and viscous damping coefficient of Equations (30) and (31)), to get:

(68) $$m_{A}=\frac{\pi }{4}\rho_{f}w^{2}\left(a_{1}+\frac{a_{2}}{w}\sqrt{\frac{2\left(\eta^{\prime }-i\eta^{\prime \prime }\right)}{\rho_{f}\omega }}\right),$$

(69) $$c_{V}=\frac{\pi }{4}\rho_{f}w^{2}\omega \left(\frac{b_{1}}{w}\sqrt{\frac{2\left(\eta^{\prime }-i\eta^{\prime \prime }\right)}{\rho_{f}\omega }}+\frac{2\left(\eta^{\prime }-i\eta^{\prime \prime }\right)}{\rho_{f}\omega }{\left(\frac{b_{2}}{w}\right)}^{2}\right).$$

Using the identity of Equation (61), $\eta^{\prime }-i\eta^{\prime \prime }=\frac{G^{\prime \prime }}{\omega }-i\frac{G^{\prime }}{\omega }$, and since $a_{1}\sim 1$ and $a_{2}\sim b_{1}$ [20] gives, after a cumbersome rearrangement [132],

(70) $$m_{A}=\frac{\pi }{4}\rho_{f}w^{2}+\frac{\pi }{2}b_{2}\frac{G^{\prime }}{\omega^{2}}+\frac{b_{1}\pi }{2\sqrt{2}}\frac{\sqrt{\rho_{f}}w}{\omega }\left(\sqrt{\sqrt{{G^{\prime }}^{2}+{G^{\prime \prime }}^{2}}+G^{\prime }}\right),$$

(71) $$c_{V}=\frac{\pi }{2}b_{2}\frac{G^{\prime \prime }}{\omega }+\frac{b_{1}\pi }{2\sqrt{2}}\sqrt{\rho_{f}}w\left(\sqrt{\sqrt{{G^{\prime }}^{2}+{G^{\prime \prime }}^{2}}-G^{\prime }}\right).$$

**Figure 9:**
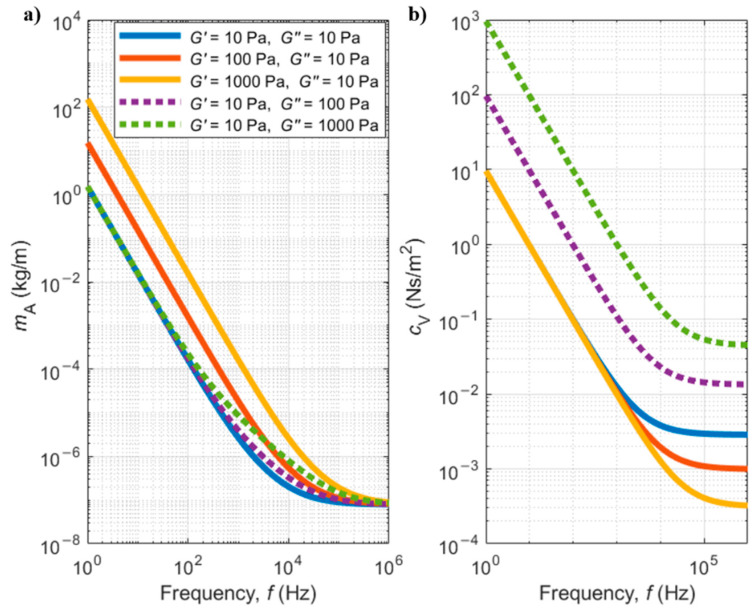
(**a**) Added mass and (**b**) viscous damping per unit length of a rectangular microcantilever (width *w* = 10 µm) as a function of the frequency of oscillation in the fluid (water, *ρ*_f_ = 1000 kg/m^3^) and of the elastic and viscous parts of the dynamic modulus, calculated with Equations (70) and (71).

Figure 9 shows plots of the added mass and viscous damping per unit length as function of the shear load frequency (corresponding to the cantilever oscillation frequency, in the case of this work), as calculated by Equations (70) and (71), for some chosen values of the elastic and viscous components of the dynamic modulus. It can be observed that the added mass is more affected by variations in the elastic part of the dynamic modulus ($G^{\prime }$) (solid red and yellow lines), while the damping coefficient is more affected by variations in the viscous term of the dynamic modulus (purple and green dashed lines) ($G^{\prime \prime }$).

Experimentally, $m_{A}$ and $c_{V}$ are obtained from the measured amplitude, H(ω), and phase, ϕ(ω), spectra using Equations (64a) and (64b), as described previously. Note that here, contrary to the method shown in the previous section, a prior knowledge of the fluid density is required, since the system of two equations is used to extract the two components of the dynamic modulus assuming a constant fluid density.

By defining the variables $B=\frac{b_{1}\pi }{2\sqrt{2}}\sqrt{\rho_{f}}w$, $C=\frac{\pi }{4}\rho_{f}w^{2}\omega ,$ and $D=\frac{\pi b_{2}}{2\omega }$, Equations (70) and (71) can be re-written as the following system of equations:

(72) $$\{\begin{array}{c} m_{A}\omega =C+DG^{\prime }+B\left(\sqrt{\sqrt{{G^{\prime }}^{2}+{G^{\prime \prime }}^{2}}+G^{\prime }}\right)\;\left(a\right)\\ c_{V}=DG^{\prime \prime }+B\left(\sqrt{\sqrt{{G^{\prime }}^{2}+{G^{\prime \prime }}^{2}}-G^{\prime }}\right)\;\left(b\right) \end{array}.$$

This system of equations can finally be solved to get the elastic and the viscous components of the dynamic modulus, $G^{\prime }$ and $G^{\prime \prime }$, respectively, as functions of the added mass, $m_{A}$, and damping coefficient, $c_{V}$ [133,134]:

(73) $$G^{\prime \prime }\left(\omega \right)=\frac{c_{V}}{D}-\frac{B}{D\sqrt{2D}}\sqrt{\sqrt{{\left(\frac{B^{2}}{D}+2\left(m_{A}\omega -C\right)\right)}^{2}+4c_{V}{}^{2}}-\frac{B^{2}}{D}-2\left(m_{A}\omega -C\right)},$$

(74) $$G^{\prime }\left(\omega \right)=\frac{1}{D}\left(m_{A}\omega -C-\frac{B^{2}G^{\prime \prime }}{c_{V}-DG^{\prime \prime }}\right).$$

**Figure 10:**
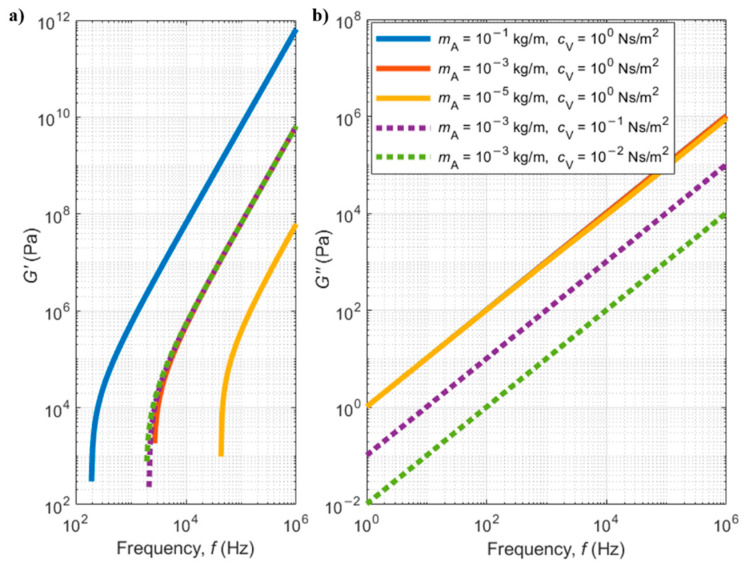
(**a**) Elastic and (**b**) viscous components of the dynamic modulus of a rectangular microcantilever (width *w* = 10 µm) as a function of the frequency of oscillation in the fluid (water, *ρ*_f_ = 1000 kg/m^3^) and of the added mass and viscous coefficient, calculated with Equations (73) and (74).

The two components of the dynamic modulus, calculated with Equations (73) and (74), are shown in Figure 10 as functions of the shear load frequency, and for some representative values of added mass and damping coefficient. In agreement with Figure 9, the elastic part of the dynamic modulus ($G^{\prime }$) mostly depends on the added mass (solid blue and yellow lines), while the viscous term of the dynamic modulus ($G^{\prime \prime }$) depends on the damping coefficient (purple and green dashed lines). It is also important to note that $G^{\prime }\sim \omega^{2}$ and $G^{\prime \prime }\sim \omega^{1}$, as predicted by the Maxwell model, and that $G^{\prime }\to 0$ in the limit of low frequencies (viscous fluid). Therefore, the fluid behaves like a viscous liquid when $G^{\prime \prime }$ dominates and starts entering the viscoelastic regime when the values of $G^{\prime }$ approach those of $G^{\prime \prime }$ [133].

The dynamic viscosity can be calculated from Equations (73) and (74) using $\left|\eta^{*}\right|={\left({\eta^{\prime }}^{2}+{\eta^{\prime \prime }}^{2}\right)}^{\frac{1}{2}}=\frac{1}{\omega }{\left({G^{\prime }}^{2}+{G^{\prime \prime }}^{2}\right)}^{\frac{1}{2}}$. One of the greatest advantages of this method is the large range of frequencies that it can cover, since the microcantilever can be designed to operate and probe high-frequency viscoelastic fluid behavior that is not accessible by conventional rheometry [133].

## 6. Outlook and Further Challenges

This work presents a thorough analysis and discussion of the role of microcantilevers in mass and rheology sensing. Despite the great progress reported in the last two decades, many challenges, both theoretical and practical, remain to be tackled before unlocking the full foreseen potential for these microdevices.

Overcoming these challenges will likely require a complementary progress in both the fundamental understanding of the dynamical response of the microcantilever oscillating and changing mass in dissipative media and of the practical implementations of sensors based on microcantilevers.

Concerning the first set of challenges, a better understanding of the physical origins of noise, both intrinsic to the microcantilever or induced by the measuring apparatus, is required. As pointed out in Section 3.3.4, the noise mechanisms are yet not fully understood, and the current practices for measuring noise may need to be rethought and standardized among the community. This would enable the development of benchmarks and metrics that can be used to better compare different techniques and tailor them to specific applications. In addition, the development of more complete models and the achievement of a deeper understanding of the dynamical non-linear response of the cantilever vibrating in a viscous medium are essential to fully exploit the behavior of non-linear systems and propose novel sensing approaches. Hysteresis, bifurcations, chaos, and energy transfer between coupled modes are only some of the effects that can arise from this highly non-linear system and that can be explored in applications. The dynamic interaction between the oscillating cantilever and a non-Newtonian viscoelastic fluid is still not well studied but has the potential of filling the gap between low-frequency characterization of these fluids (via bulk rheometry) and high-frequency measurements (via Brillouin or quartz resonators). Extract important information about the viscoelastic properties of the fluid may revolutionize point-of-care diagnostics and food processing. Extending our knowledge of the dynamic behavior in the presence of a non-zero flow velocity could also stimulate adoption in other emerging fields, such as in flow chemistry. Finally, fundamental knowledge of the kinetics of chemical reactions and optimal operation conditions that can be used for coating the beam or detecting new analytes is required.

Regarding the second set of challenges, one may mention the expected progress in microfabrication processes and materials, which can contribute to decrease costs and make high-volume industrial applications accessible, to develop new opto-electromechanical or biological functionalities to the microcantilever, or even allow the integration of sensors in curvilinear or complex three-dimensional (3D)-shaped surfaces. New geometries have started to emerge thanks to the enhanced fabrication techniques that are available today—see, for example, hollow cantilevers [135] or probes with integrated fibre optics for sensing [136]. Additionally, interfacing the beam with a proper circuitry, and integration with complementary metal-oxide-semiconductor (CMOS), is equally a crucial step for any commercial applications that will allow us to probe higher frequencies and ever-reducing time and space scales, in real-time, contributing actively to the promised next revolution of smart cities/homes and the Internet of Things (IoT), where common spaces can be filled with sensing devices that continuously monitor the environment and communicate with one another or with people.

## List of Symbols (in Order of Appearance in the Text)

L	cantilever length
w	cantilever width
h	cantilever thickness
t	time
x	space coordinate (distance from the cantilever support)
q(x,t)	time-varying distributed load acting on the beam at a distance *x* from the support, per unit length
W(x,t)	time-varying deflection of the beam at a distance *x* from the support
$F_{z}$	shear forces acting on the element of the beam
$M_{y}$	bending moment acting on the element of the beam
ρ	density of the structural material
A	area of rectangular beam cross section
$I_{z}$	second moment of area of the rectangular cross section beam
E	Young’s modulus of the structural material
ψ(t)	temporal term solution of harmonic oscillation
Φ(x)	spacial term solution of harmonic oscillation
$c_{1,2,3,4}$	constants of spacial term solution of harmonic oscillation
$f_{0,n}$	natural (undamped) resonance frequency of mode *n*
$\omega_{0,n}$	natural (undamped) radial resonance frequency of mode *n*
z	displacement of the one-degree-of-freedom microcantilever from the equilibrium position (*z* = 0)
$\dot{z}$	velocity of the one-degree-of-freedom microcantilever
$\ddot{z}$	acceleration of the one-degree-of-freedom microcantilever
$k_{\mathrm{eff}}$	effective spring constant of the microcantilever
$m_{\mathrm{eff}}$	effective mass of the *n*th resonant mode of the microcantilever
$m_{c}$	total mass of the microcantilever
c	intrinsic viscous damping coefficient
Q	quality factor
ω	excitation frequency
$F_{0}e^{i\omega t}$	excitation harmonic force at ω, with amplitude $F_{0}$
$A_{0}$	amplitude of the motion at ω
ϕ	phase between the applied external force and the motion at ω
$\omega_{res}$	resonance frequency of the *n*th mode of intrinsically damped resonators
$m_{0}\;$	mass of the cantilever per unit length
$c_{0}$	intrinsic viscous damping coefficient per unit length
$F_{hydro}\left(x,t\right)$	time-varying distributed hydrodynamic load, acting on the beam at a distance *x*, per unit length
$m_{A}$	added mass by interactions with the surrounding fluid, per unit length
$c_{V}$	added damping coefficient by interactions with the surrounding fluid, per unit length
$\omega_{R,n}$	resonance frequency of the *n*th mode of extrinsically damped resonators with added mass and damping
$Q_{n}$	quality factor of the *n*th mode
$\Gamma_{rect}{}^{'}\left(\omega \right)$	real part of the hydrodynamic load acting on a microcantilever with rectangular cross section
$\Gamma_{rect}{}^{\prime \prime }\left(\omega \right)$	imaginary part of the hydrodynamic load acting on a microcantilever with rectangular cross section
$\rho_{f}$	density of the fluid
η	viscosity of the fluid
δ	thickness of the layer in which the velocity of the fluid drops by a factor of 1/*e*
Re	Reynolds number
*a*_1_, *a*_2_, *b*_1_, *b*_2_	Maali’s constants for $\Gamma_{rect}\left(\omega \right)$
τ	integration time
$\sigma_{y}\left(\tau \right)$	Allan deviation for time windows of duration *τ*
$f_{i}$	consecutive *i*th frequency measurements
$f_{c}$	nominal carrier frequency
$S_{y}\left(f\right)$	spectral density of frequency fluctuations
$S_{\Phi }\left(f\right)$	spectral density of phase fluctuations
$y_{rms}\left(f\right)$	measured root mean squared (rms) value of normalized frequency
$\Phi_{rms}\left(f\right)$	measured root mean squared (rms) value of normalized phase
BW	width of the frequency band in Hz
$P_{noise\;\left(1\;Hz\right)}\left(f\right)$	power density in one single sideband due to phase modulation by noise, for a 1 Hz bandwidth (dBm/Hz)
$P_{signal\;}$	total power of the carrier (dBm)
ℒ(f)	single-sideband phase noise, the ratio of $P_{noise\;\left(1\;Hz\right)}\left(f\right)$ to $P_{signal\;}$(dBc/Hz)
$f_{h}$	cut-off frequency of an infinitely sharp low-pass filter
$h_{-2}$, $h_{-1}$, $h_{0}$, $h_{1}$, $h_{2}$	constants to fit power-laws to random walk frequency noise, flicker of frequency, white frequency noise, flicker of phase and white phase noise, respectively
A, B, C, D, E	numerical constants for conversion between frequency (spectral densities) and time (Allan deviation) domains
$\delta f_{min}$	minimum measurable frequency shift
LoD	limit of detection
$\delta f_{0}$	shift in the natural (undamped) resonance frequency
$\delta f_{R}$	shift in the damped resonance frequency of microcantilevers with added mass and damping;
$\delta k_{\mathrm{eff}}$	infinitesimal change of the effective stiffness of the cantilever induced by the adsorbate
$\delta m_{\mathrm{eff}}$	infinitesimal change of the effective mass of the cantilever induced by the adsorbate
$\delta m_{A}$	infinitesimal change of the added mass induced by the fluid
δη	infinitesimal change in the viscosity of the fluid
$\delta \rho_{f}$	infinitesimal change in the density of the fluid
S	sensitivity
$S_{mass,vac}$, $S_{mass,fluid}\;$	mass sensitivity in vacuum and in fluid
$S_{viscosity,fluid}$	viscosity sensitivity
$\tau_{A}$, ${\dot{\tau }}_{A}$	applied shear stress and shear stress rate
$\delta_{D}$, ${\dot{\delta }}_{D}$	shear strain and shear strain rate of a viscous dashpot
$\delta_{S}$, ${\dot{\delta }}_{S}$	shear strain and shear strain rate of an elastic spring
$\delta_{tot}$, ${\dot{\delta }}_{tot}\;$	shear strain and shear strain rate of the spring-dashpot series
$G_{0}$	elasticity constant of the fluid
λ	characteristic relaxation time of the fluid
ω	frequency of the applied shear stress and induced total strain response
φ	phase between applied stress and total strain response
$\tau_{0}$	amplitude of the shear stress
$\delta_{0}$	amplitude of the total strain response
$G^{*}$	dynamic elastic modulus
$G^{\prime }$, $G^{\prime \prime }$	elastic and viscous parts of the dynamic elastic modulus
$\eta^{*}$	complex dynamic viscosity
$\eta^{\prime }$, $\eta^{\prime \prime }$	viscous and elastic parts of the dynamic viscosity
$\left|\frac{H\left(\omega \right)}{H_{0}}\right|$	general ratio of amplitudes of the transfer function
